# The PreKit platform: Cryptic gene clusters activation and high-titer compound production

**DOI:** 10.1016/j.synbio.2026.04.007

**Published:** 2026-05-01

**Authors:** Zhongyu Chen, Yelin Duan, Lei Liu, Xiaozheng Wang, Meifeng Tao, Zhiyong Li, Tingting Huang, Shuangjun Lin

**Affiliations:** a State Key Laboratory of Microbial Metabolism, Joint International Research Laboratory on Metabolic & Developmental Sciences, School of Life Sciences & Biotechnology, Shanghai Jiao Tong University, 800 Dongchuan Road, Shanghai, 200240, China; b Haihe Laboratory of Synthetic Biology, Tianjin, 300308, China; c Frontiers Science Center for Transformative Molecules, Shanghai Jiao Tong University, Shanghai, 200240, China; d Hainan Research Institute, Shanghai Jiao Tong University, Sanya, 572025, China

**Keywords:** PreKit, Toolkit, Heterologous expression, Cryptic gene clusters activation, High-titer compound production

## Abstract

The discovery of novel scaffold compounds or enzymes through genome mining of biosynthetic gene clusters (BGCs) represents cornerstone of drug development. A major obstacle, however, is that most microbial BGCs encoding secondary metabolites remain silent under standard laboratory conditions. While promoter engineering coupled with heterologous expression has become a mainstream strategy to activate these silent BGCs, its efficiency is often limited by inherent incompatibilities among key factors, including host selection, promoter strength, and culture medium. To overcome this issue, we developed a streamlined platform integrating a promoter-library with an *indC*-based reporter system. This platform enables combinatorial screening across diverse media conditions and different heterologous hosts, systematically resolving the multi-factor incompatibility issue. Building on this strategy, we further developed a plug-and-play promoter toolkit, which was successfully applied to activate a silent type II polyketide synthase (PKS) gene cluster (*spa*). This led to the production of one new aromatic polyketides compounds strepanthenes A (**1a**) and streptoketide C (**1b**). Furthermore, enhancement of precursor supply was achieved by integrating a non-carboxylative malonyl-CoA (NCM) pathway into *S. lividans* LJ1018 through *attP*^TG1^ site-specific integration system, we achieved a 3.6-fold and 3.3-fold increase in the titers of compounds **1a** and **1b**, respectively. Collectively, this work provides a versatile and efficient strategy for activating silent BGCs and improving metabolite production. Bioactivity evaluation revealed that new compound **1a** exhibited antibacterial activity against *S. aureus* and *E. faecalis*, with a consistent MIC value of 4 μg mL^−1^ against both strains.

## Graphical abstract

**Figure:**
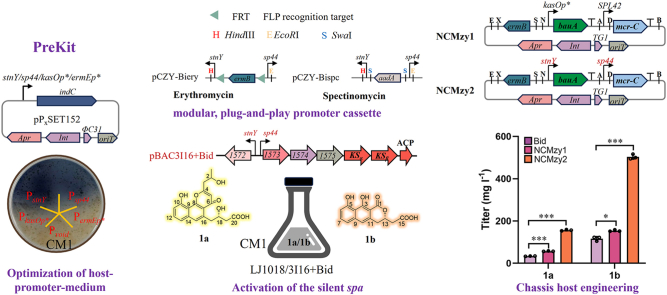

## 1 Introduction

Microbial natural products exhibit a broad spectrum of potent bioactivities, spanning antimicrobial, antiviral and anticancer properties, underscoring their immense potential in drug development. As indispensable sources of pharmaceutical leads and aproved drugs, these metabolites hold a prominent position in innovative drug discovery [1]. Recent advances in whole-genome sequencing have enabled rapid and accurate acquisition of complete genomic datasets from marine-derived microorganisms, including *Streptomycetes*. Bioinformatics platforms like antiSMASH [2] and PRISM [3] allow precise identification of biosynthetic gene clusters (BGCs) encoding key enzymes responsible for producing diverse structural scaffold, including nonribosomal peptide synthetases (NRPSs), polyketide synthases (PKSs), terpenoids, and ribosomally synthesized and post-translationally modified peptides (RiPPs). To access the metabolites encoded by these BGCs, researchers have developed several in situ strategies for activating silent BGCs. These approaches include the OSMAC [4] (one strain many compounds), which leverages variable cultivation conditions to elicit metabolite production; overexpression of PPTase (phosphopantetheinyl transferase) to activation of PKS-derived pathways [5]; targeted induction of pathway-specific positive regulators [6,7]; application of chemical elicitors (HiTES) [8] to stimulate secondary metabolite production; and promoter engineering [[9], [10], [11], [12]] to fine-tune the expression of key biosynthetic genes. However, the feasibility of these genetic manipulation strategies varies drastically across distinct microbial hosts, restricting their universal applicability.

Heterologous expression [10,[13], [14], [15]] provides distinct advantages for activating silent BGCs, including targeted activation, facile genetic manipulation, and shorter fermentation cycles. For instance, cosmids [16], fosmids [17], and notably bacterial artificial chromosome (BAC) [18] libraries enables the cloning of large genomic fragments from *Streptomyces* species and other microorganisms with minimal sequence overlap. Alternatively, advanced methods such as TAR [19], ExoET [20], and CAPTURE [21] permit direct and precise capture of target BGCs for the assembly of recombinant shuttle vectors. Heterologous expression has demonstrated significant potential in enhancing the production of valuable compounds, as exemplified by the increased titer of fidaxomicin [15] or lycopene [22] achieved through multi-host screening and multi-media optimization. Despite these advances, heterologous expression remains hindered by two major bottlenecks: intrinsically low transcriptional activity of target BGCs in the foreign host, and incompatibility between the exogenous cluster and the host's native regulatory or metabolic networks. To mitigate the former, researchers commonly replace the native promoters within the target BGCs with strong, constitutive promoters [11,12]. Nevertheless, intrinsic incompatibilities among the heterologous hosts, engineered promoters, and culture media can still impede successful expression, often resulting in suboptimal metabolite yields.

Here, we report an integrated platform termed PreKit (promoter and reporter-guided selection of heterologous host fermentation media kit), which resolves the aforementioned multi-factor incompatibility by the combinatorial promoter engineering with an *indC*-based [12] reporter system. This platform enables compatibility screening across multiple heterologous hosts and diverse fermentation media. Application of this platform successfully activated the silent type II PKS gene cluster *spa*, leading to the production of strepanthene A (**1a**) and streptoketide C (**1b**) [23,24]. To further elevate the titers of **1a** and **1b**, we engineered the chassis strain *S. lividans* LJ1018 [18] by integrating the non-carboxylative malonyl-CoA (NCM) [25] pathway under the control of the optimal promoter identified via the PreKit platform screening. This metabolic engineering strategy established synergistic coupling between precursor supply and biosynthetic gene cluster expression, yielding in a 3.6-fold and 3.3-fold improvements in the titers of the two aromatic polyketides (**1a**,**1b**), respectively.

## 2 Materials and methods

### 2.1 Strains, plasmids, and primers

All primers, bacterial strains and plasmids used and constructed in this study have been summarized in Supplementary Tables S1 and S2, respectively. *Escherichia coli* DH5α was used for all plasmid construction and amplification. KOD one™ PCR Master Mix DNA Polymerase (Toyobo, Japan) for gene amplification and ClonExpress Ultra One Step Cloning Kit for plasmid construction (Vazyme, China). 2 × T5 Super PCR Mix for PCR verification (Tsingke, China). All oligonucleotides were synthesized by Tsingke or BioSune. All chemicals, including analytical standards, were of ≥98% purity. Other chemicals were purchased from Macklin (Shanghai, China) unless stated otherwise.

### 2.2 BAC library screening

The BAC library of *Streptomyces* sp. S52B was constructed using the BAC vector pMSBBAC2 [26] by Eight Star Bio-tech Company (http://www.eightstarsbio.com). Two pairs of specific primers 1513testF1/R1 and 1629testF1/R1 used for screening the BAC library were listed in Supplementary Table S1. The PCR condition is as follows: 98 °C for 5 min; 30 cycles each of 98 °C for 10 s, 60 °C for 10 s and 72 °C for 20 s; 72 °C for 2 min by using 2 × T5 Super PCR Mix with 5% DMSO. The BAC pBAC3I16 were determined to harbor the complete *spa* cluster by end-sequencing.

### 2.3 Media screening for PreKit

The hosts *S. albidoflavus* J1074 (formerly *S.albus* J1074), *S. coelicolor* M1154, *S. lividans* GX28, and *S. lividans* LJ1018-pP*_stnY/sp44/kasOp∗/ermEp∗_*SET152 (harboring multi-promoter and *indC*-based reporter) used in this study were listed in Supplementary Table S2. These host strains were obtained through biparental conjugation, as described in Supplementary section “Construction of PreKit strain”, and were typically cultured on MS medium (2% soybean powder, 2% mannitol, 2% agar, pH 7.2) medium for 5–7 d for sporulation. Aerial mycelia (approximately 0.5 cm^2^) were aseptically collected from each recombinant hosts and uniformly streaked onto over 20 optimized solid media (Supplementary Table S3) plates in a predefined array and cultivated for 5 to 7 days. Indigoidine production in CM1–CM5 liquid media from recombinant hosts, was measured by detecting OD_600_ of the 10-fold diluted supernatant of fermentation cultures using DMSO [12].

### 2.4 Construction of heterologous expression promoter insertion mutant strain

The *E. coli* strain DH5α/pBAC3I16+Bid, which harboring bidirectional promoter (*stnY-sp44*) refactoring [27] *spa* BGC was heterologously expressed in four hosts *S. albidoflavus* J1074, *S. coelicolor* M1154, *S. lividans* GX28, and *S. lividans* LJ1018 (GX28 Δ*wblA*) [18] through triparental conjugation induced by *E. coli* ET12567/pUB307. Similarly, the *E. coli* strain DH5α/pBAC3I16+Bidke, which harboring bidirectional promoter (*kasOp∗-ermEp∗*) refactoring *spa* BGC was only heterologously expressed in *S. lividans* LJ1018. Briefly, the donor strain *E. coli* DH5α/pBAC3I16+Bid or DH5α/pBAC3I16+Bidke and the helper strain *E. coli* ET12567/pUB307 were prepared by growth to an OD_600_ value of 0.6−0.8 in LB medium at 37 °C, 220 rpm. Then, cells were washed twice and resuspended in LB medium with a concentration of ∼5 × 10^9^ cells mL^−1^. The spores of four hosts *S. albidoflavus* J1074, *S. coelicolor* M1154, *S. lividans* GX28, and *S. lividans* LJ1018 were resuspended in TSB with a concentration of ∼3 × 10^6^ colonies mL^−1^, respectively. After heating at 50 °C for 10 min, the spore suspension was incubated at 30 °C for 2 h to give recipient strain. Finally, the donor strain, helper strain, and recipient strain were mixed with a ratio of 1:1:2 and spread on MS medium plate containing 20 mM MgCl_2_ [11]. After incubation at 30 °C for 16–20 h, the plate was covered with 50 μg mL^−1^ of apramycin and 50 μg mL^−1^ of trimethoprim, and incubated at 30 °C for 5−7 days. The conjugants were identified by diagnostic PCR with test primers 1513testF1/R1 a nd 1629testF1/R1 (Supplementary Table S1). Three positive clones (*n* = 3 biological replicates) were randomly selected for small scale fermentation and subjected to metabolite analyses by HPLC. HPLC and LC-MS analysis was carried out on a reverse-phase column (ACE C18, 5 μm, 250 × 4.6 mm, Avantor) with UV detection at 254 nm on a Waters H-CLASS PLUS or Waters QDa Detector under the following program: solvent A, H_2_O (0.1% formic acid); solvent B, ACN; 5% B (0–3 min), 5% to 100% B (3−33 min), 100% B (33−38 min), 5% B (38.01−40 min); flow rate at 0.5 mL min^−1^.

### 2.5 Construction of heterologous expression KS_αβ_ gene disruption mutant strain S. lividans LJ1018/3I16+BidKO

The *E. coli* strain DH5α/pBAC3I16+BidKO (Supplementary Table S2), which harboring bidirectional promoter (*stnY-sp44*) refactoring and *KS*_*αβ*_ gene disruption *spa* BGC was heterologously expressed into host *S. lividans* LJ1018 via triparental conjugation induced by *E. coli* ET12567/pUB307 as described above, and the conjugant plate was covered with 50 μg mL^−1^ of apramycin, 100 μg mL^−1^ spectinomycin, and 50 μg mL^−1^ of trimethoprim. The conjugants were identified by diagnostic PCR with test primers 3I16KO-YZF/R (Supplementary Table S1). Three positive clones (*n* = 3 biological replicates) were randomly selected for small-scale fermentation and subjected to metabolite analyses by HPLC, as described the program detailed above.

### 2.6 Metabolic analysis of host strains harboring spa BGC through small-scale fermentation

The host strains harboring bidirectional promoter (*stnY-sp44*) refactoring *spa* BGC used in this study were listed in Supplementary Table S2 was cultured on an MS medium agar plate at 30 °C for 5−7 days. Then the mycelia of these strains were inoculated into 50 mL of TSB medium (1.7% tryptone, 0.3% soy peptone, 0.5% NaCl, 0.25% K_2_HPO_4_, 0.25% glucose, pH 7.3) at 30 °C. After growing at 220 rpm for 2−3 days in a 250 mL Erlenmeyer flask, 5 mL of seed cultures was transferred to a 250 mL Erlenmeyer flask containing 50 mL of CM1/CM2/CM5/CM3/CM4 medium (Supplementary Table S3) and inoculated at 30 °C, 220 rpm for 5 days. Subsequently, the fermentation cultures were centrifuged (4000 *g*, 20 min) to yield the supernatant. The compounds in the supernatant were absorbed by resin XAD-16 (4%), and then eluted with 20 mL of methanol. After evaporation, the residues were dissolved in 200 μL of MeOH and filtered for HPLC analysis, as described the program detailed above.

### 2.7 Large-scale fermentation of S. lividans LJ1018/3I16+Bid, extraction, and isolation of metabolites

Similarly, the host strains *S. lividans* LJ1018/3I16+Bid was cultured on an MS medium for sporulation. Then the mycelia of the strains were inoculated into TSB medium and 50 mL of seed cultures was transferred to a 2 L Erlenmeyer flask containing 500 mL of CM1 medium (1.5% oat, 0.0001% FeSO_4_, 0.0001% MnCl_2_, 0.0001% ZnSO_4_, pH 7.2–7.4), A total of 45 L cultures were prepared and inoculated at 30 °C, 220 rpm for 5 days. The crude extract (25 g) was obtained by processing the supernatant that collected through centrifugation with resin XAD-16 (4%, 1800 *g*) absorption, followed by repeated elution using 10 L of methanol and subsequent concentration under vacuum. The crude extract was subjected to a silica gel column and sequentially eluted with CH_2_Cl_2_/CH_3_OH to afford six fractions (Fr1 to Fr6). Fr5 was further purified by semipreparative HPLC with the monitoring wavelength at 254 nm to yield strepanthene A (**1a**, 12 mg) and streptoketide C (**1b**, 8 mg).

### 2.8 Measurements of malonyl-CoA using the ELISA kit

The hosts *S. lividans* LJ1018:NCMzy1/2, cells were collected through centrifugation at 6000 *g* for 10 min at 4 °C at the logarithmic growth phase and the stationary phase of CM1 fermentation. The cells were then resuspended in 2 mL of ice-cold PBS buffer, and the cell wall was destroyed through ultrasonication. The suspensions were then centrifuged at 8000 *g* for 10 min at 4 °C. The malonyl-CoA concentrations in the supernatant were measured using a malonyl-CoA ELISA kit (Fankew, Shanghai Kexing Trading Co., Ltd.) for microbes according to the manufacturer's instructions. Absorbance at 450 nm was converted to concentration via standard curve and normalized to cell dry weight (CDW) [25,28].

### 2.9 Construction of S. lividans LJ1018/3I16+Bid:NCM mutant

pLTGe + NCMzy1/2 (Supplementary Table S2) were transferred into *E. coli* ET12567/pUZ8002, which as the donor strains mixed with *S. lividans* LJ1018/3I16+Bid, respectively, and spread on MS medium plate containing 20 mM MgCl_2_. After incubation at 30 °C for 20 h, the plate was covered with 200 μg mL^−1^ of erythromycin, 50 μg mL^−1^ of apramycin and 50 μg mL^−1^ of trimethoprim, and incubated at 30 °C for 5−7 days. The conjugants were identified by diagnostic PCR with test primers *ermB*-YZF and NCM-YZR (Supplementary Table S1). Three positive clones (*n* = 3 biological replicates) were randomly selected for small scale fermentation and subjected to metabolite analyses by HPLC, as described the program detailed above.

### 2.10 Construction of KS_αβ_ gene complementation mutant strain

The gene complementation vector pLTGess + *KS_αβ_* using ClonExpress assembly to ligate the *Bam*HI/*Not*I-digested pLTGess vector with the *KS_αβ_* fragment amplified by primers ess*KS*_*αβ*_F/R. And further pLTGess + *KS_αβ_* were transferred into *E. coli* ET12567/pUZ8002, which served as the donor strains and mixed with *S. lividans* LJ1018/3I16+BidKO and spread on MS medium plate containing 20 mM MgCl_2_. The conjugants were identified by diagnostic PCR with test primers *ermB*-YZF/NCM-YZR (Supplementary Table S1). Three positive clones (*n* = 3 biological replicates) were randomly selected for small scale fermentation and subjected to metabolite analyses by HPLC, as described the program detailed above.

## 3 Results

### 3.1 Promoter and reporter-guided selection of heterologous host fermentation media kit (PreKit)

**Fig. 1:**
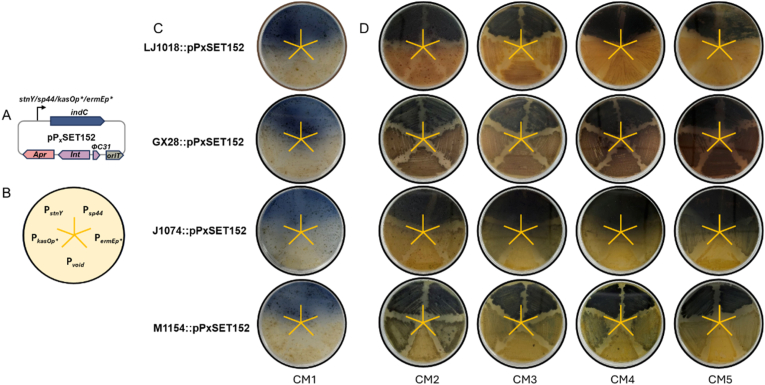
Promoter and *indC*-based reporter-guided selection of heterologous host fermentation media kit (PreKit). (A) Schematic diagram of the *indC*-based reporter-guided vector harboring promoter *stnY/sp44/kasOp∗/ermEp∗*. (B) Schematic diagram of fermentation positions of host strains harboring different promoters on solid media plate. (C) Profiling of promoter activity via solid media plate fermentation.

To address the inherent unpredictable in the compatibility among hosts, promoters, and media during heterologous expression [13], we developed a systematic strategy to evaluate the combinatorial compatibility of these key variables. Based upon on our previous work, the promoter activity order from highest to lowest (*stnY* > *sp44* > *kasOp∗* > *ermEp∗*) was confirmed in both *S.* *albidoflavus* J1074 and *S.* *lividans* TK24 hosts using the *indC*-based reporter gene system. Furthermore, qRT-PCR analysis demonstrated that under the *stnY* promoter, the precursor peptide-encoding gene *ymA* exhibited the highest transcriptional level at 24 h in both hosts, thereby effectively promoting high production of YM-216391 [12] (Fig. 1A). Accordingly, a series of reporter vectors harboring the *indC* reporter gene under the control of these distinct promoters were similarly employed to develop the PreKit platform in this study.

These reporter vectors were individually introduced into four host strains *S. albidoflavus* J1074, *S. coelicolor* M1154, *S. lividans* GX28 [18], and *S. lividans* LJ1018 (GX28 Δ*wblA*) [18] (Supplementary Table S2). The recombinant strains were subsequently inoculated onto designated positions (Fig. 1B) on a series of over 20 optimized solid media plates for incubation.

Promoters-hosts-media compatibility was directly correlated with observed differences in colony pigmentation across solid media, providing a straightforward and effective approach for preliminary screening. Following comparative analysis, over 20 optimized media were consolidated and deduplicated, all of which were compiled from the literature and have been successfully used for the heterologous expression of diverse gene clusters. It was determined that the four host strains exhibited optimal cross-compatibility with five distinct media: CM1, CM2, CM3, CM4, and CM5 (Supplementary Table S3). Concretely, visual inspection of culture plates (Fig. 1B–D) revealed consistently deeper blue pigmentation of indigo in regions expressing constructs harboring *stnY-indC* or *sp44-indC*, compared to those expressing *kasOp∗-indC or ermE∗-indC*. These results indicate that the promoters *stnY* and *sp44* promoters consistently exhibited higher activity across multiple host-medium combinations.

More specifically, on CM1 solid medium plate, all four host strains harboring the *stnY* and *sp44* promoters consistently and significantly produced a pronounced blue indigo pigment (Fig. 1C). Similarly, on CM2–CM5 solid media, these constructs also exhibited deeper coloration compared with those containing the *kasOp∗* and *ermE∗* promoters, although the hue varied from the blue indigo (Fig. 1D). This color variation may result from media components inducing endogenous pigments simultaneously in hosts such as *S. lividans* GX28 and *S. lividans* LJ1018, or from differences in light reflection owing to the intrinsic coloration of the media (Fig. 1D). Crucially, these variations do not compromise the accuracy or reliability of the PreKit platform in identifying functional promoter-host-medium combinations. To provide quantitative validation and minimize subjectivity associated with color-based visual assessment, indigoidine production was quantitatively measured spectrophotometrically. The quantitative results confirmed the visual trends, showing that constructs driven by *stnY* and *sp44* promoters produced markedly higher pigment levels than those under *kasOp∗* and *ermEp∗* control. Notably, the latter two promoters did yield indigoidine, their very low yield resulted in an extremely faint blue color, which could be easily overlooked through visual inspection alone (Supplementary Fig. 1).

Thus, the PreKit platform successfully identified five effective host-promoter-medium combinations. Among these, the hosts-*stnY*/*sp44*-CM1 combinations were prioritized for subsequent heterologous expression trials of the target gene clusters, owing to their robust and visually distinct production of the blue indigo pigment and minimal interference from endogenous pigments formation.

### 3.2 The modular, plug-and-play uni- and bidirectional promoter cassette

The genetic organization within BGCs is structured into specific transcriptional architectures, primarily unidirectional polycistronic operons (47%) or bidirectional/divergent promoter arrangements [29]. These conserved transcriptional architectures embody specific regulatory logics, whthin bacterial promoters typically facilitate an “all-or-nothing” activation of the entire biosynthetic pathway. In support of this functional principle, previous studies have demonstrated that both native unidirectional intragenic promoter (p1) and synthetic bidirectional promoters (e.g., *kasOp∗*–*kasOp∗*) refactored BGCs were successfully expressed in the commonly used model host *S. albidoflavus* J1074, yielded two peptidyl deazapurine natural products [10] or four fidaxomicin derivatives [11].

**Fig. 2:**
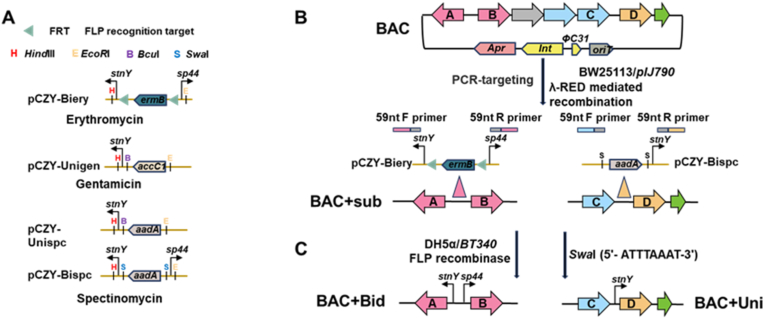
Schematic workflow for using the modular plug-and-play uni/bidirectional promoter cassette. (A) Modular plug-and-play uni/bidirectional promoter cassette harboring multi-selection resistance markers. (B) Schematic diagram of constructing BAC + sub by inserting *stnY*-*FRT-ermB-FRT-sp44* cassette or *stnY-Swa*I-*aadA-Swa*I cassette into BAC. (C) Schematic diagram of constructing BAC + Bid or BAC + Uni from BAC + sub through resistance marker excision.

Guided by the optimal promoter-host-medium combinations identified through PreKit screening, *stnY* and *sp44* showed consistently stronger activity than *kasOp∗* (Supplementary Fig. 1). We constructed a series of modular, unidirectional and bidirectional promoter cassettes using the pET28a backbone (Supplementary Fig. 2). These cassettes incorporated either *stnY* or *sp44* promoter paired with one of three resistance markers: erythromycin for pCZY-Biery, gentamicin for pCZY-Unigen, and spectinomycin for pCZY-Unispc and pCZY-Bispc (Fig. 2A). Preservation of restriction enzyme recognition sites within the modular cassettes ensures flexibility for the iterative introduction of stronger or alternative promoters in future application.

For both bidirectional or unidirectional transcriptional architectures, these plug-and-play promoter cassettes were amplified from pCZY-Biery or pCZY-Bispc and inserted between A and B or C and D, respectively (Fig. 2B), to construct BAC + sub via PCR targeting [27]. Subsequently, the resistance marker was removed either through FLP recombinase-mediated [27] excision or *Swa*I restriction digestion, yielding the final constructs designated BAC + Bid and BAC + Uni, respectively (Fig. 2C). Based on this modular framework, a total of five distinct promoters can be sequentially integrated into the BGC through the stepwise introduction of three different resistance markers. The promoters within this toolkit were designed to be fully interchangeable, thereby enabling precise fine-tuning of BGCs expression. This interchangeable modularity further facilitates the rapid optimization of heterologous biosynthetic pathways.

### 3.3 From in silico prediction to experimental activation attempts: targeting natural product BGCs in Streptomyces sp. S52B

Marine-derived microbial natural products feature novel chemical scoffolds and remarkable structural diversity [30]. This study employed the sponge-associated strain *Streptomyces* sp. S52B (Supplementary Fig. 3A) [31], which was isolated from the a South China Sea (5–10 m depth). The whole genome of *Streptomyces* sp. S52B (S52B) fully sequenced and analyzed using anti-SMASH [2] online platform, revealing BGCs predicted to encode natural products spanning a broad range of structural classes (Supplementary Table S4). In our previous work, ammosamides A and B were isolated and purified through large-scale fermentation of S52B in SSY medium [31]. Notably, the BGC (*amm*) responsible for ammosamides biosynthesis, located between BGC 13 and BGC 14, could not be predicted by antiSMASH and was localized on the S52B genome by BLAST alignment using the halogenase *mbiH* as a homologous probe [32].

Since S52B harbors multiple predicted but uncharacterized NRPS and PKS BGCs in addition to *amm*, we undertook preliminary attempts to activate these silent BGCs in situ using the following methods: (1) OSMAC [4]; (2) introducing the PPTase [5] under control of *stnY*, the highest activity of the promoter *stnY* which was characterized to be the promoter with the highest activity in S52B using the *xylE* assay test [12,33] (Supplementary Fig. 3B and C); (3) specific overexpression of positive regulatory genes within clusters, again driven by the *stnY* promoter. (4) heterologous expression of the selected BGCs in model hosts. Despite screening cross a range of optimized culture media, none of these four strategies yielded significant results (Supplementary Fig. 4).

Given the S52B harboring numerous silent BGCs and the limitations of conventional activation approaches, it presents an ideal test case for evaluating the performance and robustness of the PreKit platform.

### 3.4 PreKit platform proved capable of activating the silent spa gene cluster

A detailed comparative analysis revealed that the type II polyketide synthase (PKS) gene cluster *spa* (BGC 9) from S52B display low sequence homology to characterized clusters, indicating substantial potential for the biosynthesis of new natural products. Upstream of the core scaffold gene *miniPK* within the *spa* BGC, a pair of genes (*orf1572-orf1573*) forms a bidirectional transcriptional unit separated by a 232 bp intergenic region. Promoter prediction analysis [34] confirmed the absence of native promoter within this intergenic gap, making the *spa* BGC an ideal candidate for activation using the PreKit platform strategy.

The BAC clone pBAC3I16, harboring the *spa* BGC, was identified by diagnostic PCR using specific primers 1513testF1/R1 and 1629testF1/R1 (Supplementary Table S1). Using the PCR-targeting strategy, the plug-and-play bidirectional promoter cassette wsa amplified from plasmid pCZY-Biery (Fig. 2A) and integrated into the *spa* BGC between *orf1572* and *orf1573* to generate pBAC3I16+Bid (Supplementary Fig. 5) [27]. Subsequently, pBAC3I16+Bid was introduced into *Streptomyces* hosts via triparental conjugation, yielding the corresponding recombinant strains J1074/3I16+Bid, M1154/3I16+Bid, GX28/3I16+Bid and LJ1018/3I16+Bid (Supplementary Table S2).

**Fig. 3:**
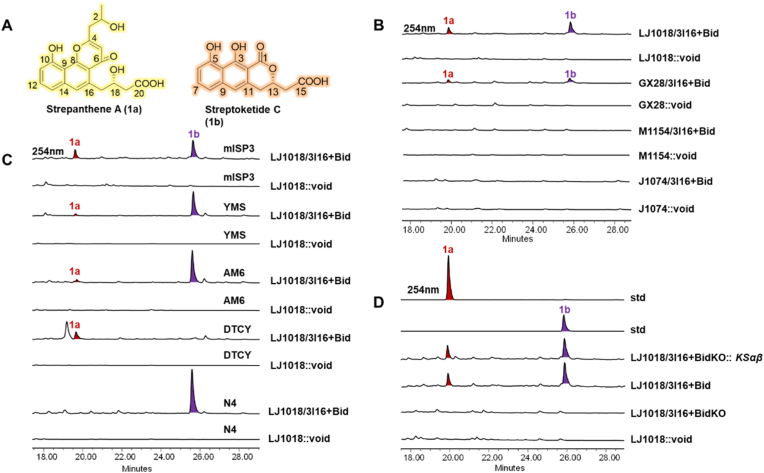
Targeted activation of the silent *spa* BGC with the PreKit platform. (A) Structure of strepanthene A (**1a**) and streptoketide C (**1b**). (B) HPLC (at 254 nm) analysis of metabolites from recombinant host J1074/M1154/GX28/LJ1018 harboring bidirectional promoter refactoring *spa* BGC cultured in CM1 medium, equally, LJ1018:void, GX28:void, M1154:void, and J1074:void were designated as recombinant host strains harboring an empty BAC vector pMSBBAC2. (C) HPLC (at 254 nm) analysis of metabolites from recombinant *S. lividans* LJ1018 harboring bidirectional promoter refactoring *spa* BGC cultured in five PreKit-screening media (CM1, CM2, CM3, CM4, and CM5). (D) HPLC (at 254 nm) analysis of standard (std) compounds **1a** and **1b** purified from 45L large-scale fermentation with metabolites from *S. lividans* LJ1018/3I16+Bid cultured in CM1 medium. LJ1018/3I16+BidKO indicates deleting the core ketoacyl synthase gene *KS_αβ_*; LJ1018/3I16+BidKO::*KS_αβ_* indicates genetic complementation of *KS_αβ_*.

These recombinants were initially cultivated individually in the highest-priority medium CM1 (Fig. 1C), as identified by the PreKit platform (Materials and Methods 2.6). Notably, the production profiles varied markedly in different recombinants under the CM1 cultivation condition. Two additional cluster-specific metabolites, strepanthenes A (**1a**) and streptoketide C (**1b**) (Fig. 3A) were detected by HPLC analysis at 254nm in the crude extract of GX28/3I16+Bid and LJ1018/3I16+Bid whereas M1154/3I16+Bid and J1074/3I16+Bid produced neither **1a** nor **1b**. The titers of **1a** and **1b** in LJ1018/3I16+Bid was approximately twofold higher than in GX28/3I16+Bid (Fig. 3B), likely attributable to delation of the global negative regulatory genes *wblA* in LJ1018 [18]. Despite the silent *spa* cluster was successfully activated using the PreKit platform, the yields of **1a** and **1b** remained suboptimal. Accordingly, four additional PreKit-identified media (CM2-CM5) were subsequently screened to identify titer enhancing conditions, with the ultimate goal of determining the optimal combination. Fermentation in CM2, CM3, and CM5 media substantially increased the titer of **1b** in LJ1018/3I16+Bid, while the production of **1a** was markedly low relative to CM1. Notably, CM4 yielded exclusively **1a**, whereas CM5 produced only **1b** (Fig. 3C), suggesting stringent nutritional control at a biosynthetic branch point. Although the precise mechanism remains to be elucidated, we hypothesize that medium composition modulates either the transcriptional regulation of the late-stage tailoring enzymes or the intracellular pool of reduced cofactors (e.g.‌, NADPH), thereby directing metabolic flux towards either congener. Future work will focus on transcriptomic comparisons between these culture conditions to identify the key regulatory switches governing this biosynthetic divergence. All these results were definitively confirmed by LC-MS analysis, with the extracted ion chromatograms (EIC) corresponding to **1a** and **1b** (Supplementary Fig. 6A and B).

These results validated the robustness of the PreKit platform, demonstrating that all screened media support the biosynthesis of either **1a** or **1b**. Collectively, through integration of the PreKit platform, plug-and-play bidirectional promoters, and HPLC-based metabolic profiling, the LJ1018-CM1 combination was identified as optimal for heterologous production of **1a** and **1b**. Accordingly, targeted deletion of the core ketoacyl synthase genes *KS_αβ_* from the promoter refactored *spa* BGC to generated pBAC3I16+BidKO (Supplementary Fig. 7). Subsequent genetic complementation of *KS_αβ_* (Supplementary Fig. 10) rigorously demonstrated that **1a** and **1b** are biosynthesized by the *spa* BGC (Fig. 3D). Compounds **1a** and **1b** (Fig. 3A and D) were isolated, purified and elucidated their structures by 1D and 2D NMR analyses (Supplementary Figs. 13–29, Tables S5 and S6) from a 45 L large-scale fermentation. Comparison of electronic circular dichroism (ECD) reported by *Qian* et al. [24] spectra of **1b** revealing C13 configuration to be *S* (Supplementary Fig. 30). The compounds **1a** and **1b** were evaluated against three Gram-positive bacteria and three Gram-negative bacteria. Compound **1a** showed the highest overall potency against *S. aureus* and *E. faecalis* compared to the positive control, with a consistent MIC value of 4 μg ml^−1^ against both test strains (Supplementary Table S7).

The plasmid pBAC3I16+Bidke, harboring the *spa* BGC refactored with *kasOp∗*/*ermEp∗* promoters, was integrated into LJ1018 to generate LJ1018/3I16+Bidke (Supplementary Fig. 8A–C). Although titers of **1a** and **1b** in LJ1018/3I16+Bidke were dramatically lower than those in LJ1018/3I16+Bid (Supplementary Fig. 8D), both these metabolites remained detectable. This result indicating that weak combinations in the PreKit screening reflect reduced productivity rather than absolute failure. These observations were definitively corroborated by LC-MS analysis (Supplementary Fig. 8E). Moreover, semi-quantitative densitometry of indigo pigmentation (Supplementary Fig. 8F) revealed a strong positive correlation with **1a** and **1b** titers, validating indigo as a reliable reporter for downstream metabolite production.

The finding that combinations scoring weakly in PreKit screening still yielded detectable metabolites confirms that PreKit minimizes false negatives and further underscores its utility as a sensitive and reliable screening platform.

### 3.5 Non-Carboxylative Malonyl-CoA (NCM) chassis host engineering boosts production titer

Aromatic polyketides represent a class of natural products with substantial industrial and pharmaceutical value [25,35]. Their biosynthesis is frequently constrained by the transcriptional regulation of the core scaffold gene *miniPK* (*KS*_*αβ*_*-ACP*) and limited availability of the key precursor malonyl-CoA [25,36]. Although replacement of the native promoter with a plug-and-play strong promoter successfully activated *spa* BGC expression, the resulting titers of two aromatic polyketides remained suboptimal. Hence, enhancing yield of these target compound represent a critical next step for elucidating and optimizing its biosynthetic pathway.

**Fig. 4:**
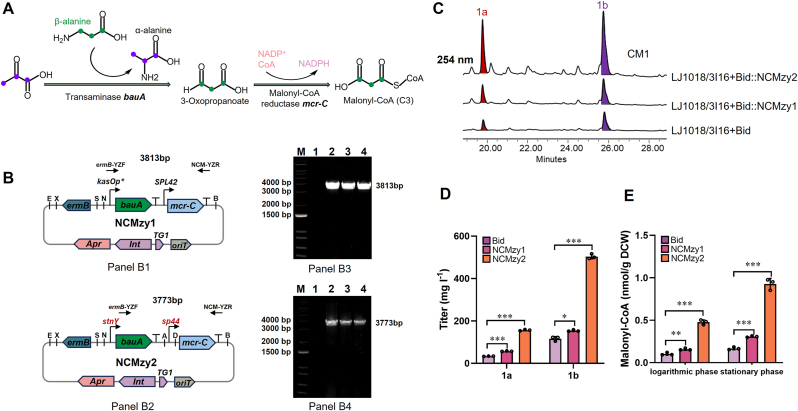
Chassis strain refactoring boosts product **1a**/**1b** titer. (A) Non-carboxylative malonyl-CoA (NCM) pathway, which converts C3–C3 via 3-oxopropanoate without carbon dioxide release, carbon loss, or ATP consumption. (B) Schematic diagram of plasmid NCMzy1 (Panel B1); Schematic diagram of optimal promoter refactored plasmid NCMzy2 (Panel B2). Panel B3/B4: PCR verification of the NCM mutant. DNA marker DL5000 (lane M); DNA templates using primers *ermB*-YZF and NCM-YZR were from the *S. lividans* LJ1018/3I16+Bid:NCMzy1/2 (NCMzy1/NCMzy2) mutant (lane 2,3,4); and the *S. lividans* LJ1018/3I16+Bid (Bid) mutant (lane 1). (C) HPLC (at 254 nm) analysis of compounds **1a** and **1b** from Bid and NCMzy1/NCMzy2. (D) The relative titers of **1a** and **1b** were evaluated by plotting normalized HPLC integrated peak. (E) Relative malonyl-CoA accumulation levels per CDW (cell dry weight) in three strains across distinct growth phases. Bar figures show mean values with error bars indicating s.d. (standard deviations, *n* = 3 biological replicates). Statistical analysis was performed using two-tailed Student's t-test (∗*P* < 0.05, ∗∗*P* < 0.01, ∗∗∗*P* < 0.0001).

The biosynthesis of **1a** and **1b** is initiated by the *miniPK* complex (Fig. 3D) that utilizes nine malonyl-CoA and one acetyl-CoA molecule as primary substrates, as inferred from the proposed biosynthetic pathway (Supplementary Fig. 11). Given the pronounced dependence of aromatic polyketide biosynthesis on malonyl-CoA supply, we aimed to augment the intracellular pool of this key precursor in heterologous hosts. Microorganisms typically generate malonyl-CoA via the acetyl-CoA carboxylase (ACC) pathway. However, this native route suffers from inherent drawbacks, including slow catalytic rates, ATP consumption, and stringent regulation by multiple intracellular metabolites [25,35,36]. Furthermore, ACC overexpression frequently induces cellular toxicity and thus compromises cell robustness [25,37]. In contrast, the non-carboxylative malonyl-CoA (NCM) pathway, a novel malonyl-CoA synthesis route recently reported by *Tan* et al. [25], proceeds via two steps: transamination (BauA) between pyruvate and β-alanine to generate 3-oxopropanoate, followed by its oxidation (MCR-C) to malonyl-CoA (Fig. 4A). This NCM pathway stably and effectively enhances malonyl-CoA flux compared to the native ACC route and has been successfully applied to boost the production of high-value polyketides including natamycin [25] and spinosad [25,36].

The plasmid pJTU6728 was integrated into the *S. lividans* SBT5 genome at the *attB*^ΦBT1^ site to generate the optimized chassis strain *S. lividans* LJ1018 [18], which has been proven to be an excellent heterologous expression host for silent BGC activation. Given that derivative strain LJ1018/3I16+Bid had already utilized both the *attB*^ΦBT1^ and *attB*^ΦC31^ integration sites, we adopted alternative site-specific integration systems for further genetic engineering [38,39]. The plasmid pLTG1 [39], harboring *attP*^TG1^ site, was selected to construct NCM [25] site-specific integration system toolkit (Supplementary Fig. 9). Using this system, two plasmid NCMzy1 (harboring promoter *kasOp∗/SPL42*) and NCMzy2 (harboring the optimal promoter *stnY/sp44*) were separately introduced into LJ1018/3I16+Bid for chassis host refactoring (Fig. 4B). The relative titers of **1a** and **1b** (three independent biological replicates per experimental group) were quantified by substituting UV absorption peak areas (Fig. 4C) into the corresponding five-point calibration curve (R^2^ ≥ 0.993) (Supplementary Fig. 12). In case of NCMzy1, the titers of **1a** and **1b** increased by only 0.7-fold (57.07 ± 1 mg l^−1^) and 0.3-fold (116.29 ± 6 mg l^−1^) (Fig. 4D); In stark contrast, using NCMzy2, the titers of **1a** and **1b** increased significantly by 3.6-fold (156.36 ± 3 mg l^−1^) and 3.3-fold (503.57 ± 10 mg l^−1^), respectively (Fig. 4D). Likewise, subsequent quantification of malonyl-CoA concentrations using an ELISA-based assay demonstrated that NCMzy2 generate significantly a higher malonyl-CoA flux than both NCMzy1 and the parental Bid strain, with 2.0-fold and 3.7-foid increases during log-phase growth, and 2.0-fold and 4.6-fold increases in stationary phase, respectively (Fig. 4E). These results collectively demonstrate that the synergistic effect between enhanced precursor supply and the optimal promoter selected from the PreKit platform effectively in boosting the high-titer production of target compounds.

## 4 Discussion

The genomic landscape of microorganism harbors millions of cryptic biosynthetic gene clusters (BGCs), representing a largely untapped reservoir of chemical diversity with immense potential for drug discovery [18]. Nevertheless, the vast majority of these BGCs remain transcriptionally silent under conventional laboratory cultivation conditions, posing a major obstacle to their functional characterization and practical exploitation. Although in situ activation of silent gene clusters offers one possible solution, its application is limited by the highly variable genetic tractability of diverse bacterial hosts. Heterologous expression provides an alternative strategy, but it also presents significant challenges [13], particularly in the selection of suitable hosts and culture media. Even when combined with promoter engineering, heterologous expression frequently suffers from low efficiency, largely because of incompatibilities among the hosts, engineered promoters, and media. In many cases, these factors are selected empirically rather than through systematic evaluation of their combined effects.

In this study, we present, for the first time, a systematic PreKit platform for screening and optimizing combinations of heterologous hosts, plug-and-play promoters, and culture media. The optimal combination identified by the PreKit platform, LJ1018–*stnY/sp44*–CM1 (host-promoter-medium), rapidly and effectively activated the silent *spa* BGC, enabling the production of the aromatic polyketide compounds **1a** and **1b**. Furthermore, we enhanced the production of **1a** and **1b** by reconstructing the chassis host through introduction of the refactored NCM [25] pathway, under optimal promote, thereby increasing the intracellular malonyl-CoA precursor supply. Based on the antibacterial activity assays, compound **1a** demonstrated potent antibacterial activity against *S.aureus* and *E. faecalis*. Collectively, these findings underscore the potential for screening and developing therapeutic agents from *streptomyces*-derived aromatic polyketides against *S. aureus*, *E. faecalis*, and other clinically relevant foodborne pathogens, thereby preventing contamination and ensuring food safety.

This study establishes a highly optimized discovery platform validated for type II PKS systems using the *spa* model. Building on this foundation, ongoing work aims to adapt the workflow to NRPS, type II PKS, and alkaloids biosynthetic systems, with the goal of extending its applicability across the broader natural product biosynthetic landscape. To address the potential limitations of the platform, we plan to implement systematic improvements in the next phase. First, the promoter library will be expanded by incorporating promoters identified in our laboratory that are stronger than *stnY* (data to be published); In parallel, chassis engineering will be pursued, inclouding the overexpression of *MbtH*-like protein [40] to boost yields of target NRPS-derived compounds, as well as the introduction PPTase to activate PKS gene clusters [5] or improve their product titers [41]; In addition, new chassis host such as ZH16NSE [42] will be further integrated, given their demonstrated ability to support the heterologous expression of cyclic peptides, type II PKS, alkaloids, and nucleoside antibiotics. Collectively, these enhancements are expected to expand the platform's capacity to activate diverse classes of biosynthetic gene clusters.

## 5 Conclusion

These findings demonstrate the rational, precise, and efficient design of the PreKit platform and highlight its promise as a powerful toolkit for activating the silent biosynthetic gene clusters. In contrast to traditional trial-and-error strategies that mainly optimize single factors, PreKit integrates three combinatorial dimensions: promoters, hosts, and media. This pre-screening strategy reduces the likelihood of choosing suboptimal conditions that result in failed or low-yield expression. As a result, crude extract analysis to be performed on only a few selected constructs, substantially streamlining the workflow. Additionally, continued optimization of PreKit platform will enable the efficient discovery of novel natural products and the production of commercially valuable compounds.

## CRediT authorship contribution statement

**Zhongyu Chen:** Writing – original draft, Visualization, Validation, Methodology, Investigation, Formal analysis, Data curation, Conceptualization. **Yelin Duan:** Investigation, Data curation. **Lei Liu:** Investigation. **Xiaozheng Wang:** Resources. **Meifeng Tao:** Resources. **Zhiyong Li:** Resources. **Tingting Huang:** Writing – review & editing, Validation, Conceptualization. **Shuangjun Lin:** Writing – review & editing, Supervision, Project administration, Funding acquisition, Conceptualization.

## Declaration of competing interest

The authors declare that they have no known competing financial interests or personal relationships that could have appeared to influence the work reported in this paper.
