# Supplementary material for: The PreKit platform: Cryptic gene clusters activation and high-titer compound production
Source: Synth Syst Biotechnol. 2026 May 1;14:171–9. doi: 10.1016/j.synbio.2026.04.007 (PMC13144596; doi:10.1016/j.synbio.2026.04.007)
Supplement: Multimedia component 1 [file mmc1.docx]

**The PreKit** **Platform: Cryptic Gene Clusters Activation and High-Titer Compound Production**

Zhongyu Chen^a^, Yelin Duan^a^, Lei Liu^a^, Xiaozheng Wang^a^, Meifeng Tao^a,b^, Zhiyong Li^a,d^, Tingting Huang^a,b*^, Shuangjun Lin^a,b,c^^,d*^

^a^ State Key Laboratory of Microbial Metabolism, Joint International Research Laboratory on Metabolic & Developmental Sciences, School of Life Sciences & Biotechnology, Shanghai Jiao Tong University, 800 Dongchuan Road, Shanghai, 200240, China.

^b^ Haihe Laboratory of Synthetic Biology, Tianjin, 300308, China.

^c^ Frontiers Science Center for Transformative Molecules, Shanghai Jiao Tong University, Shanghai, 200240, China.

^d^ Hainan Research Institute, Shanghai Jiao Tong University, Sanya, 572025, China.

Address ^*^correspondence to Tingting Huang: [tingwing82@sjtu.edu.cn](mailto:tingwing82@sjtu.edu.cn)

Address ^*^correspondence to Shuangjun Lin: [linsj@sjtu.edu.cn](mailto:linsj@sjtu.edu.cn)

**Contents**

**Experimental procedure.**

**Table S1**. Primers and oligonucleotide sequences in this study

**Table S2**. Plasmids and strains used in this study

**Table S3**. Culture Media used in PreKit

**Table S4**. Predicted biosynthetic gene clusters in *Streptomyces* sp. S52B

**Table S5**. ^1^H (700 MHz) and ^13^C (175 MHz) NMR data of strepanthene A (**1a**)

**Table S6**. ^1^H (700 MHz) and ^13^C (175MHz) NMR data of streptoketide C (**1b**)

**Table S7**. Antimicrobial activities of **1a** and **1b**

**Figure S1**. Indigoidine quantitative production in CM1-CM5 liquid media from recombinant strains

**Figure S2**. Construction of multi-selection resistance markers and uni/bidirectional promoter cassette

**Figure S3**. Promoter activity evaluation of *stnY*/*kasOp**/*ermEp** in *Streptomyces* sp. S52B

**Figure S4.** Four conventional approaches failed to activate the silent biosynthetic gene clusters in *Streptomyces* sp. S52B

**Figure S5.** The construction of *S. lividans* LJ1018/3I16+Bid mutant

**Figure S6.** Comparison of the extracted ion chromatogram (EIC) traces for compound **1a** and **1b** in different host strains and culture media

**Figure S7.** The construction of *S. lividans* LJ1018/3I16+BidKO mutant

**Figure S8.** The construction of *S.lividans* LJ1018/3I16+Bidke mutant

**Figure S9.** Schematic diagram of pLTGe+NCMzy construction

**Figure S10**. The construction of *S. lividans* LJ1018/3I16+BidKO::*KS_αβ_* mutant

**Figure S11.** Proposed biosynthetic pathway to generate the strepanthene A (**1a**) and streptoketide C (**1b**)

**Figure S12.** The standard curves of strepanthene A (**1a**) and streptoketide C (**1b**)

**Figure S13**. Structure and molecular weight of strepanthene A (**1a)**

**Figure S14**. ^1^H NMR (700 MHz) spectra of strepanthene A in DMSO-*d*_6_

**Figure S15**. ^13^C NMR and DEPT 135° NMR (175 MHz) spectra of strepanthene A in DMSO-*d*_6_

**Figure S16**. HSQC spectra of strepanthene A in DMSO-*d*_6_

**Figure S17**. ^1^H-^1^H COSY spectra of strepanthene A in DMSO-*d*_6_

**Figure S18**. HMBC spectra of strepanthene A in DMSO-*d*_6_

**Figure S19.** Structure and molecular weight of streptoketide C (**1b**)

**Figure S20.** ^1^H NMR (700 MHz) spectra of streptoketide C in DMSO-*d*_6_

**Figure S21**. ^13^C NMR and DEPT 135° NMR (175 MHz) spectra of streptoketide C in DMSO-*d*_6_

**Figure S22**. HSQC spectra of streptoketide C in DMSO-*d*_6_

**Figure S23**. ^1^H-^1^H COSY spectra of streptoketide C in DMSO-*d*_6_

**Figure S24**. HMBC spectra of streptoketide C in DMSO-*d*_6_

**Figure S25**. ^1^H NMR (700 MHz) spectra of streptoketide C in pyridine-*d*_5_

**Figure S26**. ^13^C NMR and DEPT 135° NMR (175 MHz) spectra of streptoketide C in pyridine-*d*_5_

**Figure S27**. HSQC spectra of streptoketide C in pyridine-*d*_5_

**Figure S28**. ^1^H-^1^H COSY spectra of streptoketide C in pyridine-*d*_5_

**Figure S29**. HMBC spectra of streptoketide C in pyridine-*d*_5_

**Figure S30**. Experimental electronic circular dichroism (ECD) spectra of streptoketide C (**1b**)

**Experimental procedure.**

**Activity evaluation of promoter *stnY/kasOp*/ermEp** in** ***Streptomyces*** **sp. S52B using the *xylE* assay**

The *E. coli* strain ET12567/pUZ8002 harboring pDR3-void/*stnY/kasOp*/ermEp** [1] was respectively transferred into *Streptomyces* *sp.* S52B (S52B) via biparental conjugation**.** Briefly, the donor strain *E. coli* ET12567/pUZ8002 harboring pDR3 derivatives were prepared by growth to an OD_600_ value of 0.6−0.8 in LB medium (0.5% Yeast extract, 1% tryptone, 1% NaCl) at 37 °C, 220 rpm. Then, 10 mL of cells were washed twice and resuspended in 500 μL LB medium. The recipient strain *of* S52B spores were inoculated into ISP2 medium (0.4% yeast extract, 1% malt extract, 0.4% glucose, 3% sea salt, pH 7.0) and cultured for 36 hours. Subsequently, 1 mL of the mycelial culture was harvested, washed twice, and the resulting pellet was resuspended in 500 μL of fresh ISP2 medium. Finally, the donor strain and recipient strain were mixed and spread on MS medium (soybean powder 20 g, mannitol 20 g, agar 20 g) plate containing 20 mM MgCl_2_. After incubation at 30 °C for 24 h, the plate was covered with 50 μg mL^−1^ of kanamycin and 50 μg mL^−1^ of trimethoprim, and incubated at 30 °C for 5−7 days. Then, the *xylE* (catechol 2,3-dioxygenase) activity was quantitatively measured according to a previously described method [1, 2] with minor modifications. In brief, 1 mL of cell culture was collected at various time points and centrifuged at 4000 rpm for 10 min at 4 °C. The pellet was washed three times with cold deionized water and resuspended in 1 mL of sample buffer (100 mM phosphate buffer, pH 7.5; 20 mM Na-EDTA, pH 8.0; 10% v/v acetone), followed by sonication on ice. After the addition of 0.1% Triton X-100, the samples were incubated on ice for 10 min and centrifuged at 12,000 rpm for 10 min at 4 °C. The resulting supernatant (cell lysate) was transferred to a fresh tube, and 20 μL of the lysate was mixed with 1 mL of assay buffer (10 mM phosphate buffer, pH 7.5; 0.2 mM catechol).

**Construction of PreKit strain**

These plasmids pP*_void/stnY/sp44/kasOp*/ermEp*_*SET152 [1] were transferred into *E. coli* ET12567/pUZ8002 severing as donor strains, and subsequently introduced into *S. coelicolor* M1154/*S. lividans* GX28 [3]/ *S. lividans* LJ1018 [3], respectively, via conjugation on MS agar plates supplemented with 20 mM MgCl_2_. After incubation at 30 °C for 20 h, the plates were overlaid with 50 μg mL^−1^ apramycin and 50 μg mL^−1^ of trimethoprim, and further incubated at 30 °C for 5−7 days. The conjugants were identified by diagnostic PCR.

**Construction of multi-selection resistance markers and uni/bidirectional promoter cassette**

Using the ClonExpress Ultra One Step Cloning Kit, the FRT-*aadA-oriT*-FRT fragments[4] was ligated into the restriction enzyme digested pET28a vector to construct SubVector-1. Subsequently, the *stnY* and *sp44* fragments[1] were inserted stepwise, yielding subvector-2 and subvector-3, respectively, which served as universal promoter cassettes for the cosmid or fosmid plasmid lacking *oriT*. The *accC1* fragment [5] or *aadA* fragment was ligated into the reverse linearization SubVector-2 to construct pCZY-Unigen or pCZY-Unispc. The FRT*-ermB-*FRT fragment from pJTU6722 or *Swa*I*-aadA-Swa*I fragment was ligated into the reverse linearization SubVector-3 to construct pCZY-Biery or pCZY-Bispc. The *kasOp*, ermEp*,* and *aadA* fragments was ligated into the restriction enzyme digested pET28a vector to construct pCZY-kespc. All aforementioned fragments were amplified with the primers listed in Table S1. These recombinant vector harboring uni/bidirectional promoter cassette were introduced into *E. coli* DH5α and confirming the accuracy by sequencing.

**Promoter insertion and** **gene disruption in *E. coli* BW25113/pIJ790/3I16**

The positive BAC pBAC3I16 were retransformed into *E. coli* BW25113/pIJ790 through electroporation. The promoter cassette was inserted or *KS_αβ_* genes were inactivated following the λ-RED mediated gene replacements[4]. Details for individual gene inserted or disruptions were depicted in Fig. S2–S3. The FRT-*stnY-ermB-sp44-*FRT gene cassette were amplified from plasmid pCZY-Biery with primers 3I16BiF/R (Table S1), and followed by gel purification, which were introduced into *E. coli* BW25113/pIJ790/3I16 through electroporation. The positive clones where the targeted gene was replaced by FRT-*stnY-ermB-sp44-*FRT cassette were selected with erythromycin resistance and identified by diagnostic PCR (2251 bp) using the test primers 3I16Bi-YZF/R (Table S1), and the mutant clone was transferred into *E. coli* DH5α/BT340 through electroporation. A single colony was streaked on an LB agar plate without antibiotics for single colonies and grown overnight at 42 °C to induce expression of the FLP recombinase followed by the loss of plasmid BT340[4]. Erythromycin sensitive and apramycin resistance clones indicate the successful loss of the resistance cassette and are further verified by diagnostic PCR (919 bp) using the test primers 3I16Bi-YZF/R (Table S1). The corresponding clones pBAC3I16+Bid carrying the mutated alleles were retransformed into *E. coli* BW25113/pIJ790 through electroporation. And the *aadA* were amplified from plasmid pCZY-Unispc with primers 3I16KOF/R (Table S1), and followed by gel purification, which were transformed into *E. coli* BW25113/pIJ790/3I16+Bid through electroporation. The positive clones where the targeted gene *KS_αβ_* was replaced by *aadA* were selected with spectinomycin resistance and identified by diagnostic PCR using the test primers 3I16KO-YZF/R (Table S1). Finally, pBAC3I16+Bid and pBAC3I16+BidKO were retransferred into *E. coli* DH5α through electroporation, which as the donor strains for conjugation.

**Construction and development of an** **NCM site-specific integration system toolkit**

The pLTG1[6] vector was digested with HindIII and EcoRI [restriction enzymes](https://www.sciencedirect.com/topics/agricultural-and-biological-sciences/restriction-enzyme), and the *ermB* fragments were amplified using primer TG6722F/R that were ligated into the digested vector using ClonExpress to construct recombinant vector pLTGe. Using ClonExpress, coupling SpeI and *Not*I digested pLTGe with *kasOp*-BauA-SPL42-MCR-C* fragment [7] amplified by primer NCMF/R to construct pLTGe+NCMzy1. Using ClonExpress, coupling SpeI and *BamH*I digested pLTGe with *stnY-sp44* fragment amplified by primer essF/R to construct pLTGess. Using ClonExpress, coupling (1) *Not*I and *Avr*II digested pLTGess with *BauA* fragment amplified by primer essF/R; (2) *BamH*I and *Nde*I digested (1) vector with *MCR-C* fragment amplified by primer MCRC-R/NCMF to construct pLTGe+NCMzy2. All recombinant vector harboring NCM cassette was introduced into *E. coli* DH5α and confirming the accuracy by sequencing.

**Antimicrobial assay**

The antimicrobial activities of compounds **1a**/**1b** were evaluated against three Gram-positive bacteria (*Enterococcus faecalis* ATCC29212, *Staphylococcus aureus* ATCC25923, *Sarcina lutea*), three Gram-negative bacteria (*Escherichia coli* EDL933, *Acinetobacter baumannii* ATCC19606, *Pseudomonas aeruginosa* PAO1). Ciprofloxacin was used as a positive drug of bacteria. The detailed methodologies for biological testing have been described in a previous report[8].

**Table S1**. Primers and oligonucleotide sequences in this study

| **Primers** | | **Sequence** | **Enzyme site** | |
| --- | --- | --- | --- | --- |
| **Uni/Bidirectional promoters cassette (5’-3’)** | | | | |
| 28aYZF |  | cccctcaagacccgtttaga | |  |
| 28aYZR |  | ggggaattgtgagcggataa | |  |
| 778F | | ggtggtgctcgagtgcggccgcaagcttGATGTGTAGGCTGG | | *Hind*III |
| 778R | | caaatgggtcgcggatccgaattcGATATTCCGGGGATCCGT | | *EcoR*I |
| stnYF | | tcgagtgcggccgcAAGCTTctcttcagcagtcatgtgaGCATGC | | *Hind*III |
| stnYR |  | CCAGCCTACACATCaagcttgcatccggtccgcgaaggat | | *Hind*III |
| sp44F |  | AGGTCGACGGATCCCCGGAATATCgaattctgttcacattcgaacCgtct | | *EcoR*I |
| sp44R |  | ggacagcaaatgggtcgcggatccgaattcacaccagactttacaacacc | | *EcoR*I |
| 28Rev1F | | tgttcacattcgaacCgtctctgctttgac | |  |
| 28Rev1R | | taggaagcgcctggcctacg | |  |
| 28Rev2F | | gaattcggatccgcgacccatttgc | |  |
| 6722F | | atccttcgcggaccggatgcGATATTCCGGGGATCCGTCG | |  |
| 6722R | | agacGgttcgaatgtgaacaGATGTGTAGGCTGGAGCTGC | |  |
| 778SwaIF | | ccatccttcgcggaccggatgcATTTAAATATGAGCTCAGCCAATCGACT | | *Swa*I |
| 778SwaIR |  | agagacGgttcgaatgtgaacaATTTAAATGCTGACGCCGTTGGATACAC | | *Swa*I |
| aadAF |  | atccttcgcggaccggatgcACTAGTATGAGCTCAGCCAATCGACT | | *Bcu*I |
| aadAR | | tgggtcgcggatccgaattcGCTGACGCCGTTGGATACAC | | *EcoR*I |
| accCF | | atccttcgcggaccggatgcACTAGTTTAGGTGGCGGTACTTGGGT | | *Bcu*I |
| accCR  kasOpF  kasOpR  ermEpF  ermEpR  aadAeF  aadAkR | | tgggtcgcggatccgaattcAGCTCCTGATTCCCTTTGTC  GTGTATCCAACGGCGTCAGCTGTTCACATTCGAACGGTCT  TGGGTCGCGGATCCGAATTCAACTCCCCCAGTCCTGCACG  TCGAGTGCGGCCGCAAGCTTCGCTGGATCCTACCAACCGG  AGTCGATTGGCTGAGCTCATGCGAGTGTCCGTTCGAGTGG  AGCCGCCACTCGAACGGACACTCGCATGAGCTCAGCCAATCGACT  AGCAGAGACCGTTCGAATGTGAACAGCTGACGCCGTTGGATACAC | | *EcoR*I  *EcoR*I  *Hind*III |
| **For screening BAC library (5’-3’)** | | | | |
| 1513Test-F1 |  | ATGCCCAAGATCGCACTCCTGT | |  |
| 1513Test-R1 |  | CACGTCCGCGACGAGCCGC | |  |
| 1629Test-F2 |  | ATGTCCGTGAACAGCATCCGCAC | |  |
| 1629Test-R2 |  | TCAGAGCTCCAGGCTCCACTGGG | |  |
|  |  |  | |  |
| **For amplification of homologous arms from genomic DNA for gene insertion or disruption (5’-3’)** | | | | |
| 3I16Bi-YZF | | TCACGTCGCAGGACGTGCCG | |  |
| 3I16Bi-YZR | | ACGCCGCCGTAGTGGAGGAC | |  |
| 3I16BiF | | gagctctcctccggtgggcggtcgatggccggtcggtgcctcttcagcagtcatgtgaG | |  |
| 3I16BiR |  | gatcgcccctcatgtcggacgtagccgttttcgaccgtgacaccagactttacaacacc | |  |
| 3I16KO-YZF | | catcaccgggatcggggtgg | |  |
| 3I16KO-YZR | | gacgacctggtcgcgcaggg | |  |
| 3I16KOF | | gacttcgacccgctcgaccacggcttcaccgccgaggagGCTGACGCCGTTGGATACAC | |  |
| 3I16KOR | | gacgacgtccacgtcgccgggggccagtccggcgtcggcATGAGCTCAGCCAATCGACT | |  |
|  | |  | |  |
| **For genes complementation (5’-3’)** | | | | |
| essKS_αβ_F | | CGTTGCCCTAAGCAACTGTTCCTAGCGTCACGTCAGGATCCtcagccggccggggcgc | | *BamH*I |
| essKS_αβR_ | | TGCtcacatgactgctgaagagGCGGCCGCatgaaccgacgtgtcgcca | | *Not*I |
|  | |  | |  |
| **For site-specific integration systems toolkit (5’-3’)** | | | | |
| TG6722F | | CTAGCGTCACGTCAGGATCCGCGGCCGCACTAGTGGAATCTTTAAAACAAGCAAATACA | | *BamH*I *Spe*I |
| TG6722R | | TATGACATGATTACGAATTCTCTAGATTATTTCCTCCCGTTAAATAATAGATAACTATT | | *EcoR*I *Xba*I |
| ermB-YZF | | TGAACGGGTTCACAGCGGCG | |  |
| ermB-YZR | | TGGAAAGCGGGCAGTGAGCG | |  |
| NCMF | | ACTGTTCCTAGCGTCACGTCAGGATCCTTACACGGTAATCGCCCGTCCGC | | *BamH*I |
| NCMR | | TTAAAGATTCCACTAGTGCGGCCGCGGAACGATCGTTGGCTGTGTTCACA | | *NotI* |
| NCM-YZR | | CGACCAGGCAATGTTTTCGCCT | |  |
| essF | | CTAGCGTCACGTCAGGATCCGGTACCacaccagactttacaacacc | | *BamH*I |
| essR |  | GTTTTAAAGATTCCACTAGTgcatccggtccgcgaaggat | | *Spe*I |
| BauAF | | agagacGgttcgaatgtgaacaCCTAGGTTACGCAATGCCGTTCAGCGCT | | *Avr*II |
| BauAR | | CtcacatgactgctgaagagGCGGCCGCATGAATCAGCCGCTGAACGTGG | | *Not*I |
| MCRC-R | | gcggtgttgtaaagtctggtgtCATATGATGGCAGATCTCAGCGCCACCA | | *Nde*I |
| MCRC-YZR | | TTAACGCGCGCCCGGAAGAA | |  |

The enzyme site highlighted in red was incorporated into the primer sequence during synthesis.

**Table S2**. Plasmids and strains used in this study

| Plasmid and Strain | | Features and functions | Sources and references | |
| --- | --- | --- | --- | --- |
| **Plasmid for BAC** | | | | |
| pMSBBAC2 |  | Apr, BAC library vector, ΦC31 *int/attP*, and *oriT* RK2 | | [9] |
| pBAC3I16 |  | Apr, pMSBBAC2-derived plasmid harboring entire *spa* gene cluster | | This study |
| pBAC3I16+subBid | | Apr/Ery, pBAC3I16-derived plasmid harboring promoter engineering cassette FRT-*stnY*-*ermB-sp44-*FRT | | This study |
| pBAC3I16+Bid | | Apr, pBAC3I16+subBid-derived plasmid harboring promoter engineering cassette *stnY*-*sp44* remove *ermB* resistance gene | | This study |
| pBAC3I16+BidKO  pBAC3I16+Bidke | | Apr/Spc, pBAC3I16+Bid-derived plasmid harboring *aadA* replace *KS_αβ_*  Apr/Spc, pBAC3I16-derived plasmid harboring promoter engineering cassette *kasOp**-*aadA -ermEp** | | This study  This study |
|  | |  | |  |
| **Plasmid for uni/bidirectional promoter cassette** | | | | |
| pIJ778 |  | Spc, source of *oriT* and *aadA* fragment | | [4] |
| pIJTU6722 |  | Ery, source of FRT site and *ermB* fragment | | Prof.Meifeng Tao's Gift |
| pBSPPC |  | Gen, source of *aacC* fragment | | [5] |
| pP*_stnY_*SET152 | | Apr, source of *stnY* fragment | | [1] |
| pP*_sp44_*SET152 | | Apr, source of *sp44* fragment | | [1] |
| pET28a | | Kan, vector for protein expression or used to construct the promoter engineering cassette in this work | | Novagen |
| Subvector-1 | | pET28a-derived plasmid for PCR amplification of the promoter engineering cassette FRT-*aadA-oriT-*FRT | | This study |
| Subvector-2 | | pET28a-derived plasmid for PCR amplification of the promoter engineering cassette FRT-*stnY-aadA-oriT-*FRT | | This study |
| Subvector-3 | | pET28a-derived plasmid for PCR amplification of the promoter engineering cassette FRT-*stnY*-*aadA-oriT*-*sp44-*FRT | | This study |
| pCZY-Biery |  | pET28a-derived plasmid for PCR amplification of the promoter engineering cassette FRT-*stnY*-*ermB*-*sp44-*FRT*, oriT* free | | This study |
| pCZY-Bispc |  | pET28a-derived plasmid for PCR amplification of the promoter engineering cassette *stnY*-*Swa*I-*aadA*-*Swa*I-*sp44, oriT+*FRT free, *Swa*I site insert | | This study |
| pCZY-Unispc | | pET28a-derived plasmid for PCR amplification of the promoter engineering cassette *stnY*-*aadA*, *oriT+*FRT free | | This study |
| pCZY-Unigen  pCZY-kespc | | pET28a-derived plasmid for PCR amplification of the promoter engineering cassette *stnY-aacC1*, *oriT+*FRT free  pET28a-derived plasmid for PCR amplification of the promoter engineering cassette *kasOp**-*aadA-ermEp*, oriT+*FRT free | | This study  This study |
| **All pET28a-derived plasmid constructs were transformed into *E. coli* DH5α** | | | | |
| **Plasmid for site-specific integration systems toolkit** | | | | |
| pSET152-NCM1 | | Apr, source of *kasOp*-BauA-SPL42-MCR-C* fragment, ΦC31 *int/attP*, and *oriT* RK2 | | [7] |
| pLTG1 |  | Apr, TG1 *int/attP*, and *oriT* RK2 | | [6] |
| pUCczy |  | Amp, source of *stnY-sp44* fragment, synthesized | | This study |
| pLTGe |  | Apr/Ery, pLTG1-derived plasmid harboring resistance gene *ermB* | | This study |
| pLTGess |  | Apr/Ery, pLTGe-derived plasmid harboring promoter *stnY* and *sp44* | | This study |
| pLTGe+NCMzy1 | | Apr/Ery, pLTGe-derived plasmid harboring *kasOp*-BauA-SPL42-MCR-C* fragment | | This study |
| pLTGe+NCMzy2 | | Apr/Ery, pLTGe-derived plasmid harboring *stnY-BauA-sp44-MCR-C* fragment | | This study |
| pLTGess+ *KS_αβ_* | | Apr/Ery, pLTGess-derived plasmid for *KS_αβ_* gene complementation | | This study |
| **All pLTGe-derived plasmid constructs were transformed into *E. coli* ET12567/pUZ8002** | | | | |
| ***Escherichia coli*** | | | | |
| DH5α |  | Host harboring BAC vector or general cloning | | Invitrogen |
| ET12567/pUZ8002 | | Donor strain for biparental conjugation between *E. coli* and *Streptomyces* | | Our lab |
| ET12567/pUB307 | | Donor strain for triparental conjugation between *E. coli* and *Streptomyces* | | Our lab |
| BW25113/pIJ790 | | Host strain for PCR targeting | | [4] |
| DH5α/BT340 | | Host strain for FLP recombinase mediated excision through FRT site | | [4] |
| DH5α/pBAC3I16 | | Apr, strain harboring BAC plasmid pBAC3I16 | | This study |
| DH5α/pBAC3I16+subBid | | Apr/Ery, strain harboring BAC plasmid pBAC3I16+subBid | | This study |
| DH5α/pBAC3I16+Bid | | Apr, strain harboring BAC plasmid pBAC3I16+Bid | | This study |
| DH5α/pBAC3I16+BidKO  DH5α/pBAC3I16+Bidke | | Apr/Ery, strain harboring BAC plasmid pBAC3I16+BidKO  Apr/Spc, strain harboring BAC plasmid pBAC3I16+Bidke | | This study  This study |
| DH5α/pDR3-void | | Apr, pSET152-derived plasmid harboring *xylE-neo* | | [1] |
| DH5α/pDR3-  *stnY*/*kasOp*/ermEp** | | Apr, pDR3-void-derived plasmid for the expression of *xylE* under the control of pDR3-*stnY*/*kasOp*/ermEp** | | [1] |
| DH5α/ pP_void_SET152 | | Apr, pSET152-derived plasmid harboring *indC* | | [1] |
| DH5α/ pP*_stnY/sp44/kasOp*/_*  *_ermEp*_*SET152 | | Apr, pP_void_SET152-derived plasmid for the expression of *indC* under the control of *stnY*/sp44/*kasOp*/ermEp** | | [1] |
|  | |  | |  |
| ***Streptomyces*** | | | | |
| *Streptomyces* sp. S52B | | Wild-type strain | | Prof. Zhiyong Li's Gift |
| *S. albidoflavus* J1074  *(formerly S. albus* J1074*)* | | Model *Streptomyces* host | | Our lab |
| *S. coelicolor* M1154 | | Model *Streptomyces* host | | Our lab |
| *S. lividans* GX28  *(formerly S. lividans* SBT18*)* | | Model *Streptomyces* host | | [3] |
| *S. lividans* LJ1018 | | Model *Streptomyces* host | | [3] |
| J1074::void |  | *S.albidoflavus* J1074 harboring pMSBBAC2 | | This study |
| M1154::void |  | *S.coelicolor* M1154 harboring pMSBBAC2 | | This study |
| GX28::void |  | *S.lividans* GX28 harboring pMSBBAC2 | | This study |
| LJ1018::void |  | *S.lividans* LJ1018 harboring pMSBBAC2 | | This study |
| S52B::pDR3 |  | Integration of pDR3 in *Streptomyces* sp. S52B | | This study |
| S52B::pDR3-*stnY* | | Integration of pDR3-*stnY* in *Streptomyces* sp. S52B | | This study |
| S52B::pDR3-*kasOp** | | Integration of pDR3-*kasOp** in *Streptomyces* sp. S52B | | This study |
| S52B::pDR3-*ermEp** | | Integration of pDR3-*ermEp** in *Streptomyces* sp. S52B | | This study |
| J1074-M1154-GX28  -LJ1018/3I16+Bid  LJ1018/3I16+BidKO  LJ1018/3I16+BidKO  ::*KS_αβ_*  LJ1018/3I16+Bid::NCMzy1  LJ1018/3I16+Bid::NCMzy2  LJ1018/3I16+Bidke | | Integration of pBAC3I16+Bid in four model *Streptomyces* host  Integration of pBAC3I16+BidKO in host LJ1018  Integration of pLTGess+*KS_αβ_* in LJ1018/3I16+BidKO  Integration of pLTGe+NCMzy1 in LJ1018/3I16+Bid  Integration of pLTGe+NCMzy2 in LJ1018/3I16+Bid  Integration of pBAC3I16+Bidke in host LJ1018 | | This study  This study  This study  This study  This study  This study  This study |
| **For PreKit** | |  | |  |
| J1074/M1154/GX28  /LJ1018::pP_void_SET152 | | Integration of pP_void_SET152 in J1074/M1154/GX28/LJ1018 | | This study |
| J1074/M1154/GX28  /LJ1018::pP*_stnY_*SET152 | | Integration of pP*_stnY_*SET152 in J1074/M1154/GX28/LJ1018 | | This study |
| J1074/M1154/GX28  /LJ1018::pP*_sp44_*SET152 | | Integration of pP*_sp44_*SET152 in J1074/M1154/GX28/LJ1018 | | This study |
| J1074/M1154/GX28  /LJ1018-pP*_kasOp*_*SET152 | | Integration of pP*_kasOp*_*SET152 in J1074/M1154/GX28/LJ1018 | | This study |
| J1074/M1154/GX28  /LJ1018::pP*_ermE*_*SET152 | | Integration of pP*_ermEp*_*SET152 in J1074/M1154/GX28/LJ1018 | | This study |

The BAC library of *Streptomyces* sp. S52B was constructed using the BAC vector pMSBBAC2[9] by Eight Star Bio-tech company (http://www.eightstarsbio.com).

**Table S3**. Culture media used in PreKit

| CM1 | 1.5% oat meal, 0.0001% FeSO_4_, 0.0001% MnCl_2_, 0.0001% ZnSO_4_, pH 7.2-7.4 |
| --- | --- |
| CM2 | 0.4% yeast extract, 1% malt extract, 0.4% soluble starch, 0.7% oat meal, 0.5% CaCO_3_, pH 7.3 |
| CM3 | 0.5% yeast extract, 2% soluble starch, 1% glucose, 0.5% peptone, 0.5%CaCO_3_, pH 7 |
| CM4 | 4% dextrin, 0.75% tomato paste, 0.5% yeast extract, 1% polypepetone, pH 7 |
| CM5 | 0.8% fish meal, 1.5% soluble starch, 0.5% bacterial peptone, 0.2% CaCO_3_, 0.8% glycerol, 0.02% KBr |
| CM6 | 1% fish meal, 0.5% CaCO_3_, 2% glycerol, 0.5% yeast extract, pH 7.2-7.4 |
| CM7 | 2% sucrose, 0.5% yeast extract, 2.1% Mops, 1% glucose, 0.025% K_2_SO_4_, 0.1% MgCl_2_·6H_2_O, 0.1% casamino acids |
| CM8 | 1.0% glucose, 2.0% soluble starch, 1.0% malt extract, 0.5% corn flour, 1.0% maltose, 0.2% CaCO_3_; and 1.0% trace elements including NaB_4_O_7_·10H_2_O 0.2 g/L, MnCl_2_·4H_2_O 0.2 g/L, FeCl_3_·6H_2_O 0.4 g/L, ZnCl_2_ 0.8 g/L, CuCl_2_·H_2_O 0.2 g/L, pH 7.2−7.4 |
| CM9 | 0.3% yeast extract, 0.1% trehalose, 0.1% L-proline, 0.3% beef extract, 0.6% glycerol, 0.03% K_2_HPO_4_, 0.05% MgSO_4_·7H_2_O, 0.05% FeSO_4_·7H_2_O, 0.2% CaCO_3_, pH 7.2-7.4 |
| CM10 | 0.4% yeast extract, 1% malt extract, 0.4 % glucose, pH 7.0 |
| CM11 | 2% sucrose, 1% glucose, 0.5% yeast extract, 0.5% MOPS, 0.01% casamino acids, 0.025% K_2_SO_4_, 0.1% MgCl_2_·6H_2_O |
| CM12 | 0.4 % peptone fish, 1 % starch soluble, 0.6 % corn powder, 0.2 % bacterial peptone, 0.5 % glycerol, 0.2 % CaCO_3_, pH 7.0 |
| CM13 | 1% soluble starch, 0.1% bacterial peptone, 0.05% yeast extract, 0.1% K_2_HPO_4_, 0.2% (NH_4_)_2_SO_4_, 0.1% MgSO_4_·7H_2_O, 0.1% NaCl, 0.2% CaCO_3_, pH 7.0 |
| CM14 | 0.3% yeast extract, 0.5% tryptone, 0.3% malt extract, 1% glucose, 10.3% sucrose, 2.5 M MgCl_2_·6H_2_O (2ml) |
| CM15 | 2% soluble starch, 1% polypeptone, 0.4% yeast extract |
| CM16 | 0.5% yeast extract, 0.5% polypeptone, 1% mannitol, pH 7.0 |
| CM17 | 0.4% yeast extract, 1% maltose, 0.4% glucose, 0.2% MgCl_2_, 0.2% CaCl_2_ |
| CM18 | 0.1% yeast extract, 1.5% soluble starch, 0.1% K_2_HPO_4_, 0.1% MgSO_4_, 0.2% TES, pH 7.4 |
| CM19 | 2% glucose, 0.15% KNO3, 0.28 g K_2_HPO_4_, 0.5 g MgSO_4_, 0.1 g FeSO_4_·7H_2_O, pH 7.5 |
| CM20 | 4% dextrin, 0.75% soytone, 0.5% baking yeast, 2.1% MOPS, pH 6.8 |
| CM21 | 0.8% casein peptone, 0.4% yeast extract, 0.8% MgCl_2_·6H_2_O, 5ml glycerol |

Based on media was compiled from literature used for heterologous expression of diverse gene clusters, and was rationalized by consolidating and deduplicating existing formulations.

**Table S4**. Predicted biosynthetic gene clusters in *Streptomyces* sp. S52B

| Biosynthetic gene cluster (BGC) No. | Gene cluster type | Predicted product |
| --- | --- | --- |
| BGC 1 | RiPP | aborycin |
| BGC 2 | Trans-AT PKs | unknown |
| BGC 3 | NRPS-PKs | unknown |
| BGC 4 | siderophore | gobichelin A |
| BGC 5 | NRPS | indigoidine |
| BGC 6 | / | / |
| BGC 7 | RiPP | SAL-2242 |
| BGC 8 | crocagin | ketomemicin |
| BGC 9 | ⅡPKs | unknown |
| BGC 10 | / | / |
| BGC 11 | / | / |
| BGC 12 | ⅠPKs | unknown |
| BGC 13 | NRPS | diastaphenazine |
| BGC 14 | terpene | geosmin |
| BGC 15 | NRPS-PKs | unknown |
| BGC 16 | NRPS-PEARL | unknown |
| BGC 17 | NRPS | SF2728 |
| BGC 18 | ⅠPKs | bisenarsan |
| BGC 19 | arylpolyene | cinnapeptin |
| BGC 20 | ⅠPKs | pyrrolomycin |
| BGC 21 | siderophore | kinamycin |
| BGC 22 | ⅠPKs | piericidin A1 |
| BGC 23 | RiPP | streptamidine |
| BGC 24 | / | / |
| BGC 25 | terpene | hopene |
| BGC 26 | NRPS | mirubactin |
| BGC *amm** | PEARL | ammosamides |

**amm* indicated ammosamides BGC, “/” indicated unclustered orphan BGCs

The genome of *Streptomyces* sp. S52B was sequenced and analyzed, revealing the presence of 26 natural product BGCs identified through online anti-SMASH [10] 7.1.0 (<http://antismash.secondarymetabolites.org/>). The predicted natural products encompass a diverse range of structural classes, including ribosomally synthesized and posttranslationally modified peptides (RiPPs), peptide aminoacyl-tRNA ligases (PEARLs) [11], polyketides (PKS), non-ribosomal peptides (NRPS), siderophores, and terpenes.

**Table S5**. ^1^H (700 MHz) and ^13^C (175 MHz) NMR data of strepanthene A (**1a**)

(DMSO-*d*_6_, *δ* in ppm, *J* in Hz)

| Position | *δ*_C_ | *δ*_H_ (*J* in HZ) |
| --- | --- | --- |
| 1 | 23.4 | 1.19 d (6.2) |
| 2 | 64.1 | 4.30 m |
| 3 | 42.5 | 2.81 dd (14.4, 4.3); 2.74 dd (14.4, 7.7) |
| 4 | 164.8 |  |
| 5 | 113.2 | 6.30 s |
| 6 | 178.5 |  |
| 7 | 118.0 |  |
| 8 | 156.4 |  |
| 9 | 112.8 |  |
| 10 | 154.9 |  |
| 11 | 112.2 | 7.00 d (7.7) |
| 12 | 130.5 | 7.52 t (7.9) |
| 13 | 118.0 | 7.35 d (7.9) |
| 14 | 136.7 |  |
| 15 | 127.6 | 7.48 s |
| 16 | 134.9 |  |
| 17 | 42.4 | 3.54 dd (12.5 ,4.7); 3.20 dd (12.5. 7.9) |
| 18 | 68.3 | 4.11 m |
| 19 | 42.7 | 2.34 dd (14.1, 3.6); 2.24 dd (14.8, 9.3) |
| 20 | 173.7 |  |

**Table S6**. ^1^H (700 MHz) and ^13^C (125 MHz) NMR data of streptoketide C (**1b**)

(^a^NMR data were measured in DMSO-*d*_6_. ^b^ NMR data were measured in pyridine-*d*_5_, *δ* in ppm, *J* in Hz).

| Position | | *δ*_C_^a^ | | *δ*_H_^a^ (*J* in HZ) | |  | | *δ*_C_^b^ | | *δ*_H_^b^ (*J* in HZ) | |
| --- | --- | --- | --- | --- | --- | --- | --- | --- | --- | --- | --- |
| 1 | 169.3 | |  | |  | | 170.8 | |  | |  |
| 2 | 101.2 | |  | |  | | 102.4 | |  | |  |
| 3 | 163.2 | |  | |  | | n.o. | |  | |  |
| 4 | 113.2 | |  | |  | | 115.4 | |  | |  |
| 5 | 157.1 | |  | |  | | 159.6 | |  | |  |
| 6 | 110.4 | | 6.78 brs | |  | | 111.3 | | 7.06 d (7.7) | |  |
| 7 | 131.6 | | 7.45 brs | |  | | 132.5 | | 7.52 t (7.8) | |  |
| 8 | 117.8 | | 7.19 brs | |  | | 118.5 | | 7.23 (overlapped) | |  |
| 9 | 138.9 | |  | |  | | 140.2 | |  | |  |
| 10 | 115.7 | | 7.08 s | |  | | 116.0 | | 6.91 s | |  |
| 11 | 133.4 | |  | |  | | 134.4 | |  | |  |
| 12 | 32.0 | | 3.12 m; 3.06 m | |  | | 33.7 | | 3.18 m; 3.08 m | |  |
| 13 | 75.9 | | 4.94 brs | |  | | 77.2 | | 5.34 brs | |  |
| 14 | 39.0 | | 2.83 dd (16.3, 5.0); 2.75 dd (16.2, 7.9) | |  | | 40.9 | | 3.15 m； 3.04 m | |  |
| 15 | 171.1 | |  | |  | | 172.9 | |  | |  |

**Table S7**. Antimicrobial activities of **1a** and **1b** (MIC values, μg/ml)

| strains | Gram  classification | **1a**  MIC (μg/ml) | **1b**  MIC (μg/ml) | Ciprofloxacin  MIC (μg/ml) |
| --- | --- | --- | --- | --- |
| *Enterococcus faecalis* 29212 | G^+^ | 4 | >64 | 8 |
| *Staphylococcus aureus* ATCC25923 | G^+^ | 4 | 32 | 16 |
| *Sarcina lutea* | G^+^ | 16 | 32 | 1 |
| *Escherichia coli* EDL933 | G^-^ | >64 | >64 | 1 |
| *Acinetobacter baumannii* ATCC19606 | G^-^ | >128 | >128 | 2 |
| *Pseudomonas aeruginosa* PAO1 | G^-^ | >64 | >128 | 0.25 |

Ciprofloxacin was used as the positive control for bacteria.


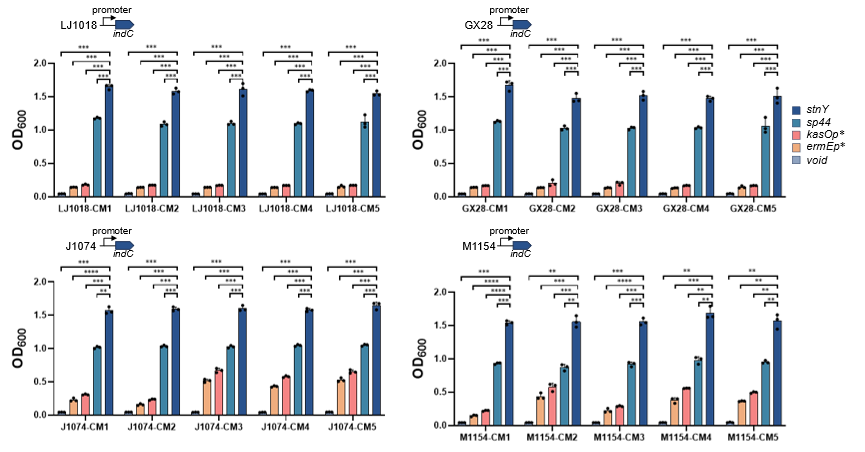


**Figure S1**. Indigoidine quantitative production in CM1-CM5 liquid media from recombinant strains. *void* indicates pP_void_SET152, *ermEp** indicates pP*_ermEp*_*SET152, *kasOp** indicates pP*_kasOp*_*SET152, *sp44* indicates pP*_sp44_*SET152, *stnY* indicates pP*_stnY_*SET152. Indigoidine production was measured by detecting OD_600_ of the 10-fold diluted supernatant of fermentation cultures with DMSO. Bar figures show mean values with error bars indicating s.d. (standard deviations, n = 3 biological replicates). Statistical analysis was performed using two-tailed Student’s t-test (***P*<0.05, ****P*<0.001, *****P*<0.0001).


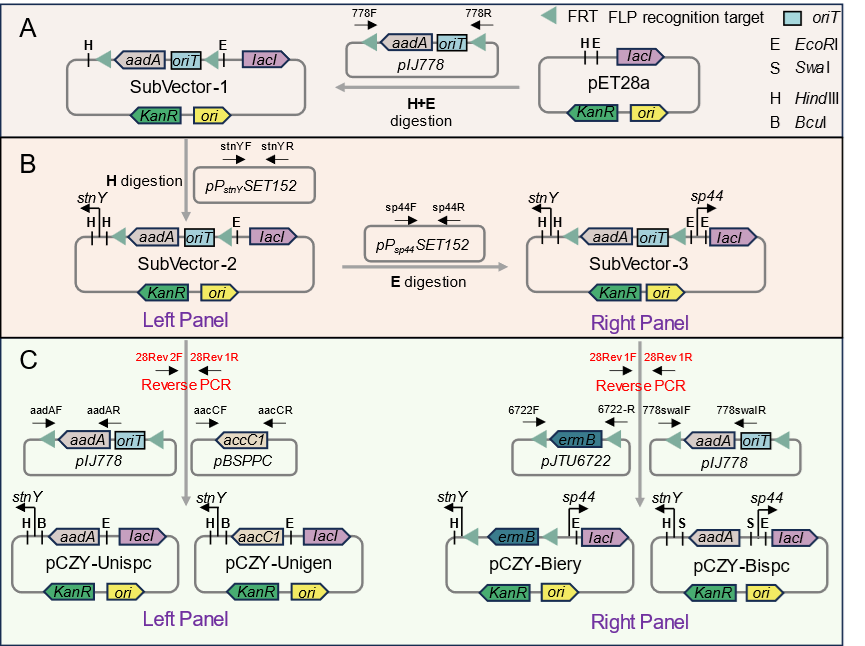


**Figure S2**. Construction of multi-selection resistance markers and uni/bidirectional promoter cassette. (A) Schematic diagram of constructing Subvector-1 where FRT-*aadA-oriT-*FRT cassette was inserted into pET28a. (B) Schematic diagram of constructing Subvector-2 (left panel) and Subvector-3 (right panel) where promoter *stnY* and *sp44* was bidirectionally inserted into Subvector-1. (C) Schematic diagram of constructing pCZY-Unispc or pCZY-Unigen by inserting *aadA* or *accC1* resistance marker into Subvector-2 (left panel). Schematic diagram of constructing pCZY-Biery by inserting FRT-*ermB-*FRT cassette into Subvector-3 to replace FRT-*aadA-oriT-*FRT cassette, and pCZY-Bispc by inserting the *SwaI-aadA-SwaI* resistance marker into Subvector-3, inserting the FLP recognition target (FRT) or enzyme cleavage site *Swa*I (5'- ATTTAAAT-3') can be used to remove the resistance marker (right panel).


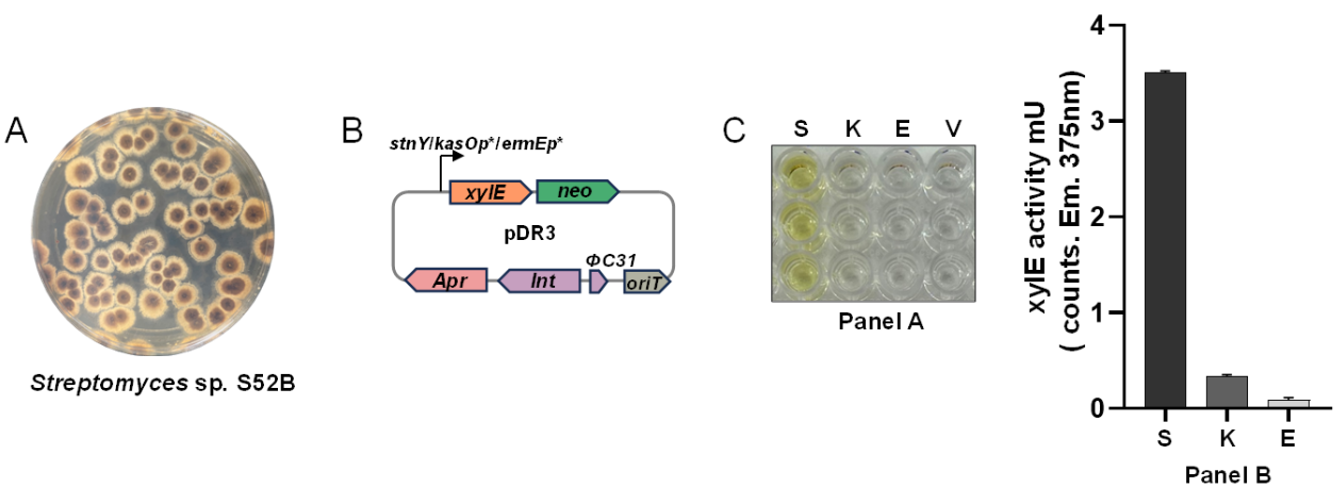


**Figure S3**. Promoter activity evaluation of *stnY*/*kasOp**/*ermEp** in *Streptomyces* sp. S52B. (A) The strain *Streptomyces* sp. S52B (S52B) displayed classic *streptomycete* morphology, characterized by the formation of sparse, pigmented aerial mycelia. (B) Schematic diagram of the reporter-guided vector pDR3 harboring promoter *stnY*/*kasOp**/*ermEp**, the pDR3-void/*stnY/kasOp*/ermEp** was transferred into *Streptomyces* sp. S52B via biparental conjugation. (C) Evaluate the activities of the promoter using the *xylE* assay, which was carried out with samples harvested after 48 h culture. Pane A/B: S52B::pDR3-*stnY* (lane S); S52B::pDR3-*kasOp**(lane K); S52B:: pDR3-*ermEp**(lane E); S52B::pDR3-void (lane V). Data represent the means ± SD of three replicates; independent experiments were repeated at least three times.

**Figure S4**. Four conventional approaches failed to activate the silent biosynthetic gene clusters in *Streptomyces* sp. S52B. We evaluated four conventional strategies for activating silent gene clusters, including OSMAC, introduction of PPTase, targeted overexpression of positive regulatory genes within the clusters, and heterologous expression without the PreKit platform, but all failed to induced activation, resulting in negative outcomes.


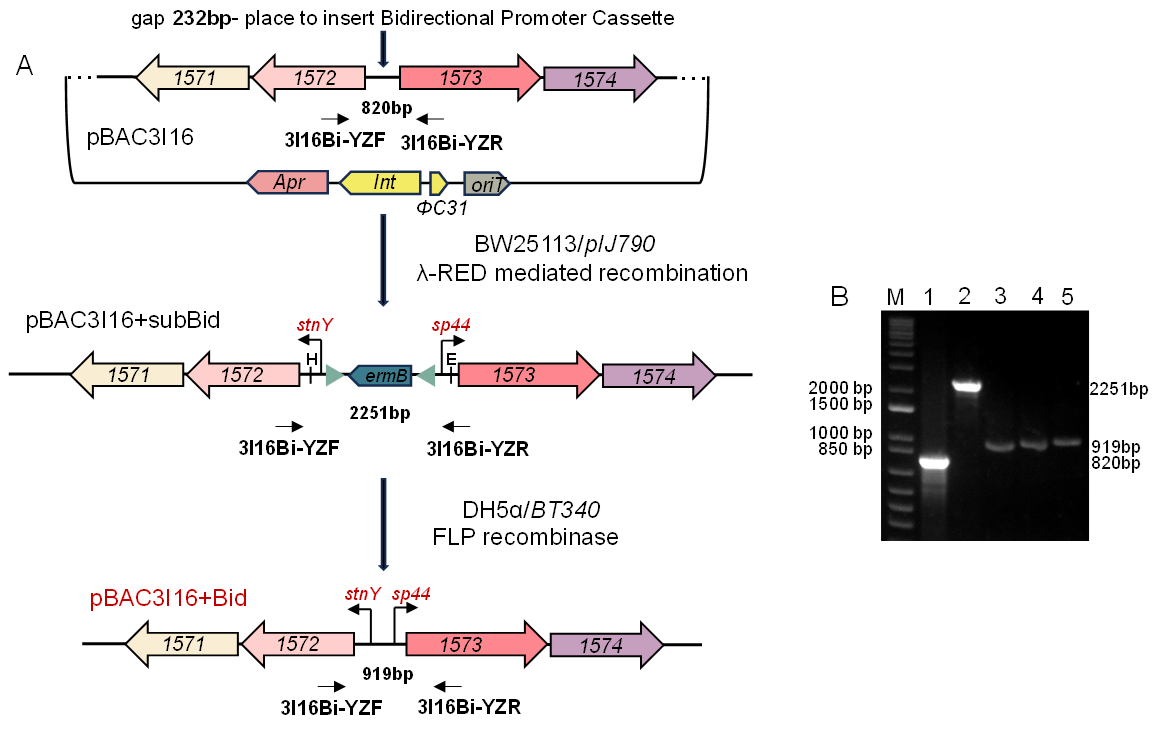
**Figure S5**. The construction of *S. lividans* LJ1018/3I16+Bid mutant. (A) The strategies used in gene in-frame insertion. Sizes of PCR products were depicted: 820 bp for the pBAC3I16, 2251 bp for the pBAC3I16+subBid, and 919 bp for the pBAC3I16+Bid, the pBAC3I16+Bid was transferred into hosts *S. lividans* LJ1018 via triparental conjugation. (B) PCR verification of the *S. lividans* LJ1018/3I16+Bid mutant. DNA marker DL5000 (lane M); DNA templates using primers 3I16Bi-YZF and R were from: the pBAC3I16 (lane 1); the pBAC3I16+subBid (lane 2); and the *S.lividans* LJ1018/3I16+Bid mutant (lane 3,4,5).


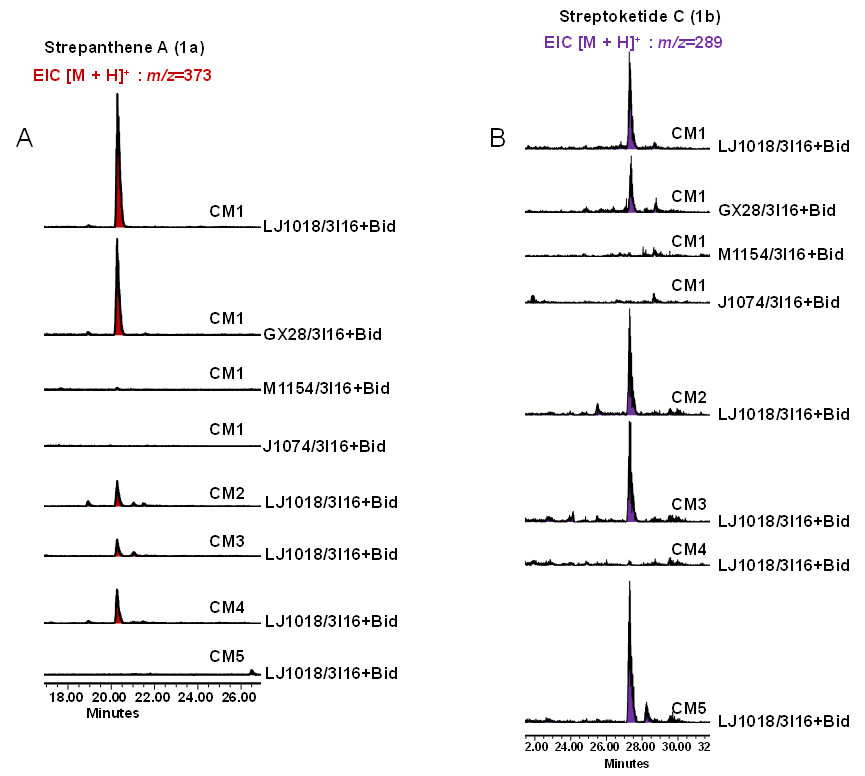


**Figure S6**. Comparison of the extracted ion chromatogram (EIC) traces for compound **1a** and **1b** in different host strains and culture media. (A) Comparison of the extracted ion chromatogram (EIC) traces for **1a** (*m/z*=373), a cluster-specific metabolite from recombinant host LJ1018/GX28 harboring bidirectional promoter refactoring *spa* BGC and culture media (CM1/CM2/CM3/CM4/CM5). (B) Comparison of the extracted ion chromatogram (EIC) traces for **1b** (*m/z*=289), a cluster-specific metabolite in host strains LJ1018/GX28 harboring bidirectional promoter refactoring *spa* BGC and culture media (CM1/CM2/CM3/CM4/CM5).


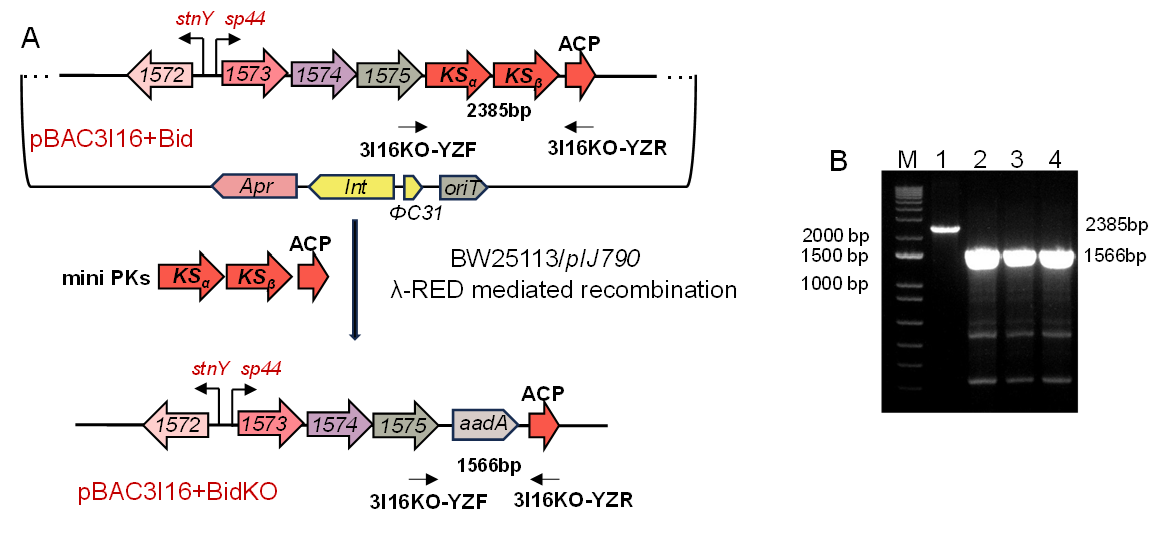


**Figure S7**. The construction of *S. lividans* LJ1018/3I16+BidKO mutant. (A) The strategies used in gene in-frame deletion. Sizes of PCR products were depicted: 2385 bp for the pBAC3I16+Bid and 1566 bp for the pBAC3I16+BidKO, the pBAC3I16+BidKO was transferred into host *S. lividans* LJ1018 via triparental conjugation. (B) PCR verification of the *S. lividans* LJ1018/3I16+BidKO mutant. DNA marker DL5000 (lane M); DNA templates using primers 3I16KO-YZF and R were from: the *S. lividans* LJ1018/3I16+BidKO mutant (lane 2,3,4); and the pBAC3I16+Bid (lane 1).


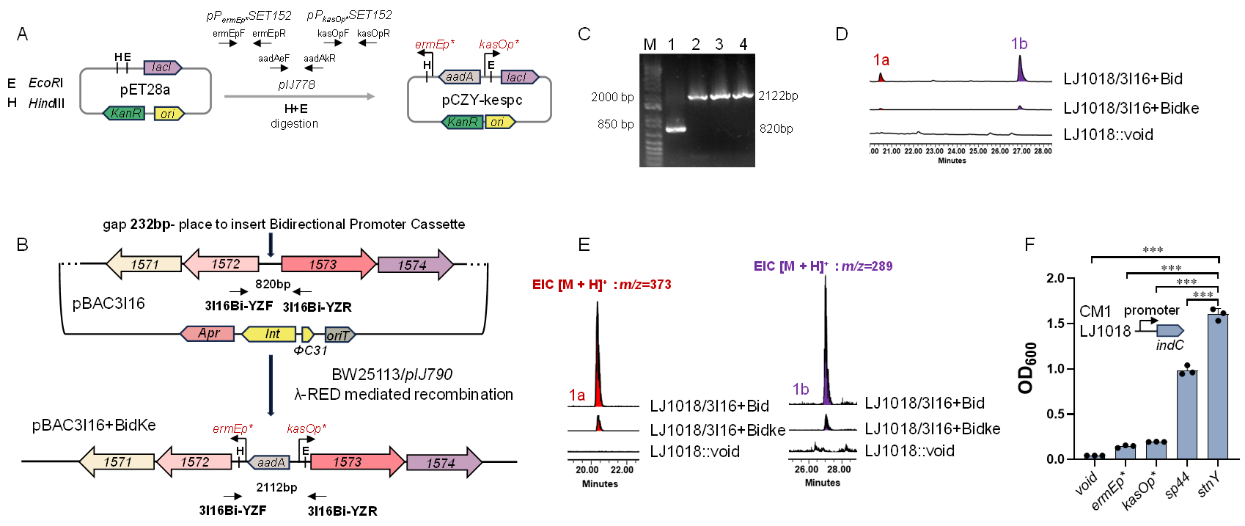


**Figure S8**. The construction of *S.lividans* LJ1018/3I16+Bidke mutant. (A) Schematic diagram of constructing pCZY-kespc where *kasOp*-aadA-ermEp** cassette was inserted into pET28a. (B) The strategies used in gene in-frame insertion. Sizes of PCR products were depicted: 820 bp for the pBAC3I16, 2122 bp for the pBAC3I16+Bidke, the pBAC3I16+Bidke was transferred into hosts *S. lividans* LJ1018 via triparental conjugation. (C) PCR verification of the *S. lividans* LJ1018/3I16+Bidke mutant. DNA marker DL5000 (lane M); DNA templates using primers 3I16Bi-YZF and R were from: the pBAC3I16 (lane 1); and the *S.lividans* LJ1018/3I16+Bidke mutant (lane 2, 3, 4). (D) HPLC (at 254nm) analysis of compounds **1a** and **1b** from recombinant strains cultured in CM1 medium. LJ1018/3I16+Bid indicates harboring *stnY-sp44* promoter refactoring *spa* BGC; LJ1018/3I16+Bidke indicates harboring *kasOp*-ermEp** promoter refactoring *spa* BGC. (E) Comparison of the extracted ion chromatogram (EIC) traces for **1a** (*m/z*=373) and **1b** (*m/z*=289). (F) Indigoidine production in CM1 liquid media from recombinant strains, void indicates LJ1018-pP_void_SET152, *ermEp** indicates LJ1018-pP*_ermEp*_*SET152, *kasOp** indicates LJ1018-pP*_kasOp*_*SET152, *sp44* indicates LJ1018-pP*_sp44_*SET152, *stnY* indicates LJ1018-pP*_stnY_*SET152. Indigoidine production was measured by detecting OD_600_ of the 10-fold diluted supernatant of fermentation cultures with DMSO. All data were represented as mean ± SD (*n* = 3 independent experiments). Statistical analysis was performed using two-tailed Student’s t-test (***, *P*<0.001).


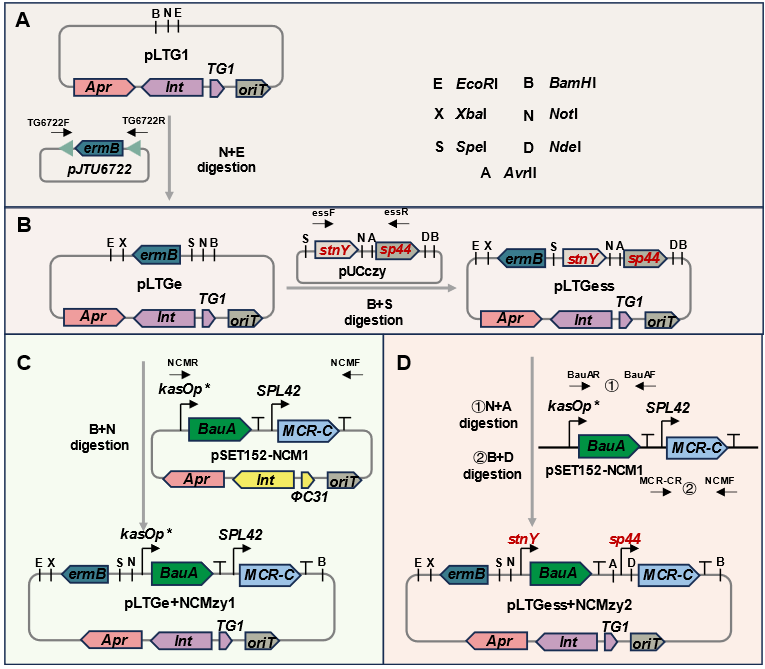


**Figure S9**. Schematic diagram of constructing pLTGe+NCMzy. (A) Schematic diagram of constructing pLTGe where *ermB* resistance marker was inserted into pLTG1. (B) Schematic diagram of constructing pLTGess where promoter *stnY-sp44* was inserted into pLTGe. (C) Schematic diagram of constructing pLTGe+NCMzy1 where *kasOp**-*BauA-SPL42-MCR-C* cassette was inserted into pLTGe. (D) Schematic diagram of constructing pLTGess+NCMzy2 where *BauA* and *MCR-C* fragment was separately inserted into pLTGess.


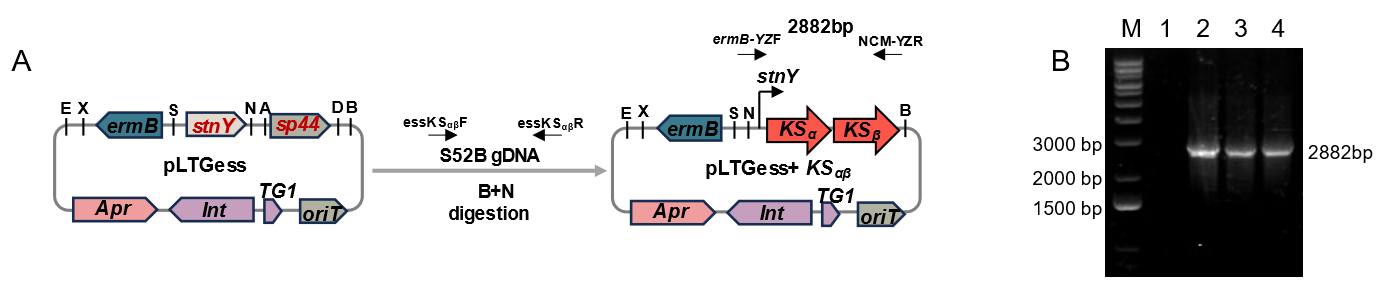


**Figure S10**. The construction of *S. lividans* LJ1018/3I16+BidKO::*KS**_αβ_* mutant. (A) Schematic diagram of constructing *KS_αβ_* gene complementation plasmid pLTGess+ *KS_αβ_*, the pLTGess+ *KS_αβ_* was transferred into hosts *S. lividans* LJ1018/3I16+BidKO via biparental conjugation. (B) PCR verification of the *KSαβ* gene complementation mutant. DNA marker DL5000 (lane M); DNA templates using primers *ermB*-YZF and NCM-YZR were from the *S. lividans* LJ1018/3I16+BidKO::*KS_αβ_* mutant (lane 2,3,4); and the *S. lividans* LJ1018/3I16+BidKO (lane 1).


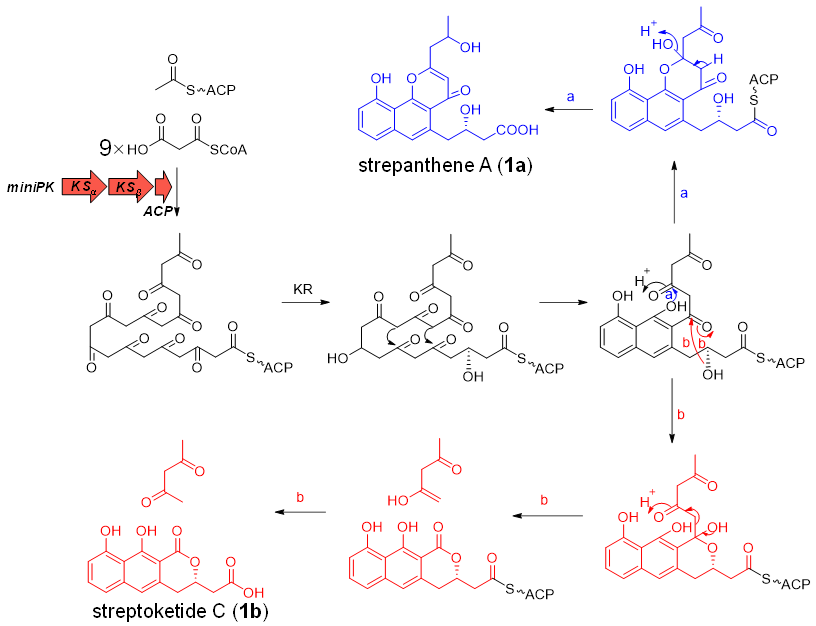


**Figure S11**. Proposed biosynthetic pathway to generate the strepanthene A (**1a**) and streptoketide C (**1b**).


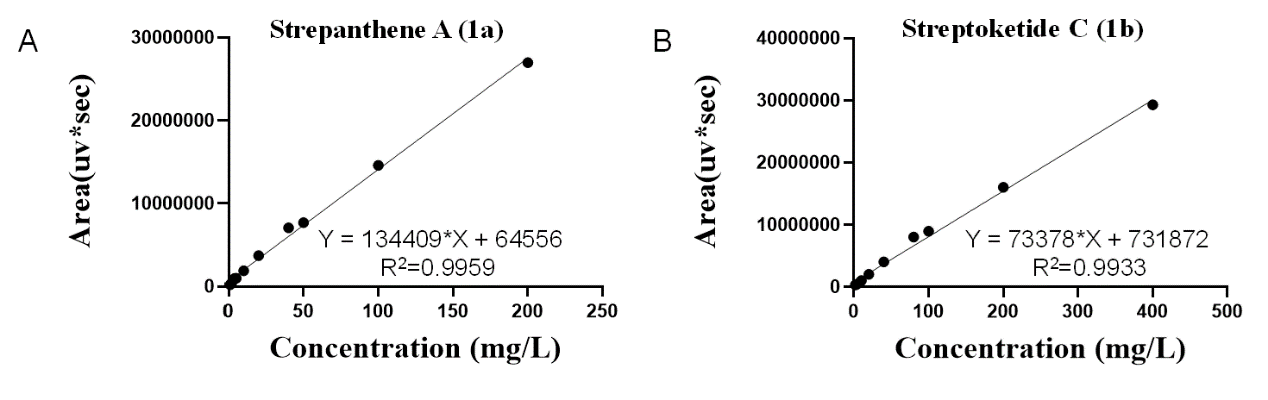


**Figure S12**. The standard curves of strepanthene A (**1a**) and streptoketide C (**1b**). (A) The standard curves used to calculate the production levels of **1a**. (B) The standard curves used to calculate the production levels of **1b**.


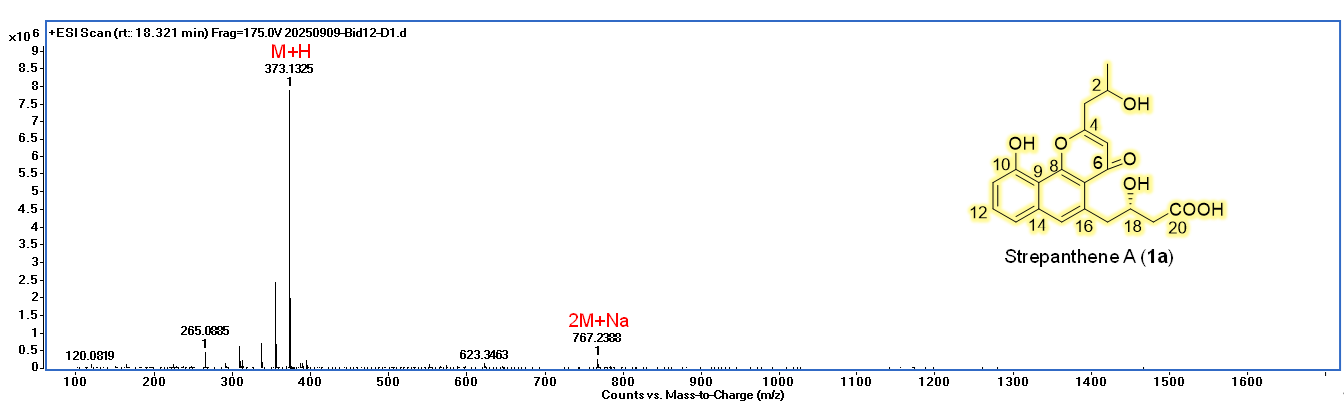


**Figure S13**. Structure and molecular weight of strepanthene A (**1a**)

**Figure S14**. ^1^H NMR (700 MHz) spectra of strepanthene A in DMSO-*d*_6_

**Figure S15**. ^13^C NMR and DEPT 135° NMR (175 MHz) spectra of strepanthene A in DMSO-*d*_6_

**Figure S16**. HSQC spectra of strepanthene A in DMSO-*d*_6_

**Figure S17**. ^1^H-^1^H COSY spectra of strepanthene A in DMSO-*d*_6_

**Figure S18**. HMBC spectra of strepanthene A in DMSO-*d*_6_

**Figure S19**. Structure and molecular weight of streptoketide C (**1b**)


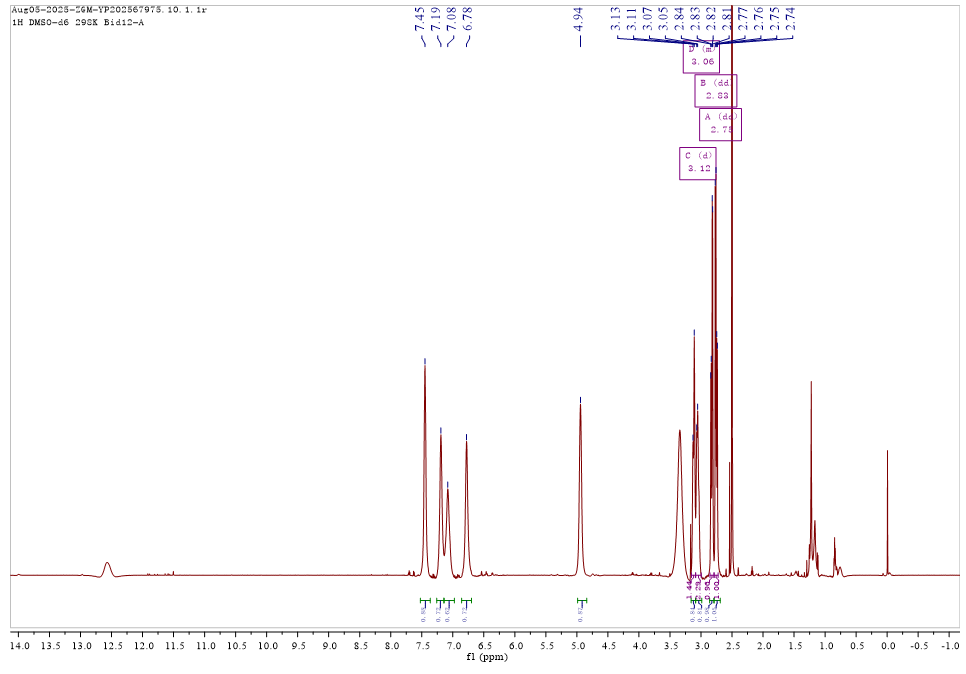


**Figure S20**. ^1^H NMR (700 MHz) spectra of streptoketide C in DMSO-*d*_6_


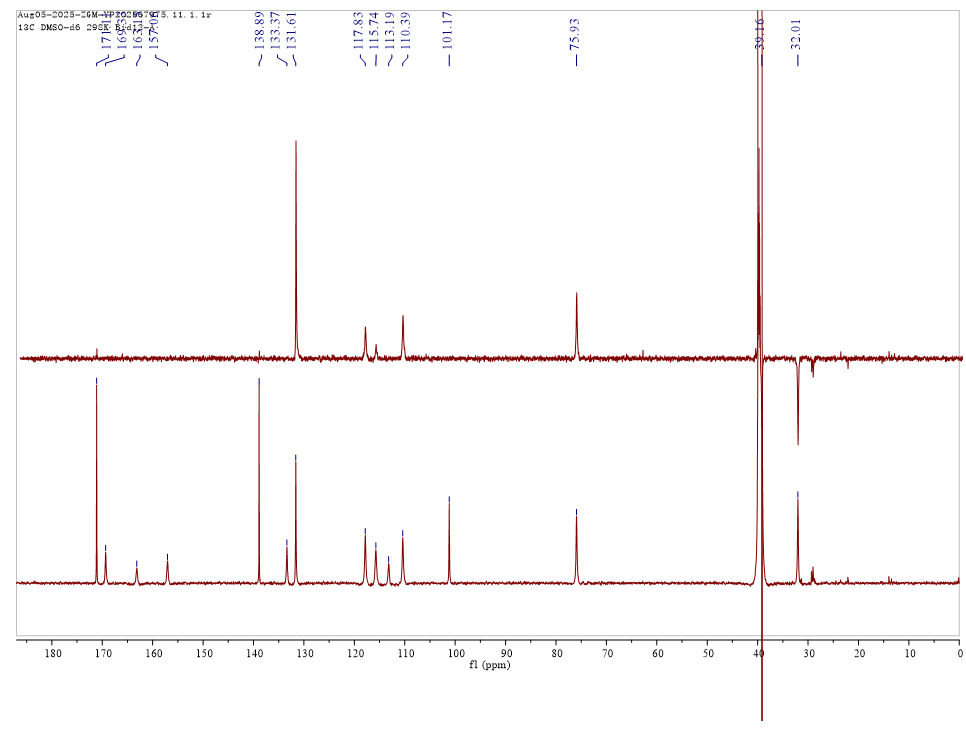


**Figure S21**. ^13^C NMR and DEPT 135° NMR (175 MHz) spectra of streptoketide C in DMSO-*d*_6_


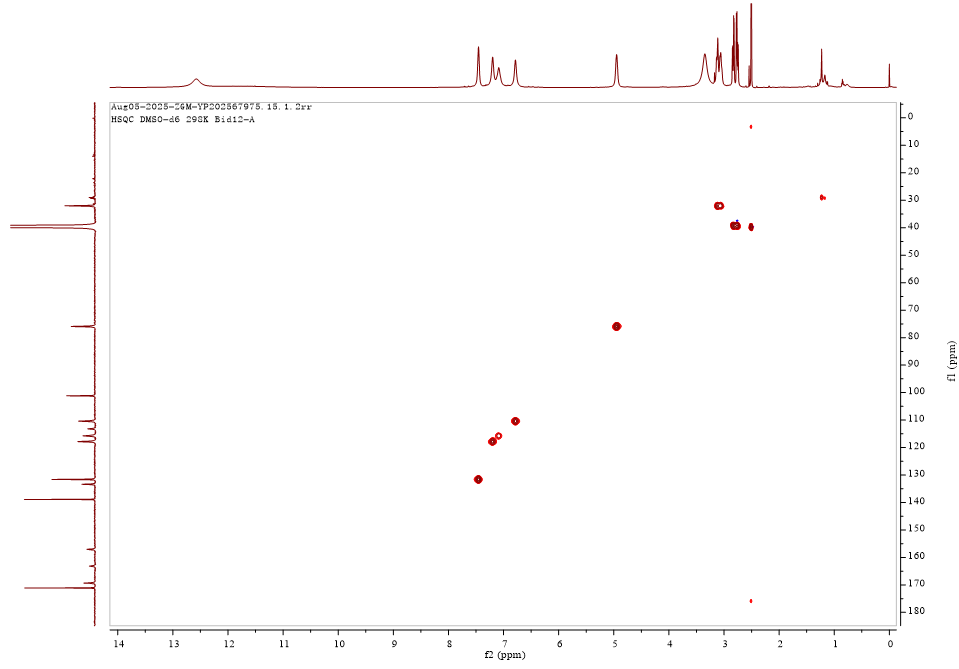


**Figure S22**. HSQC spectra of streptoketide C in DMSO-*d*_6_


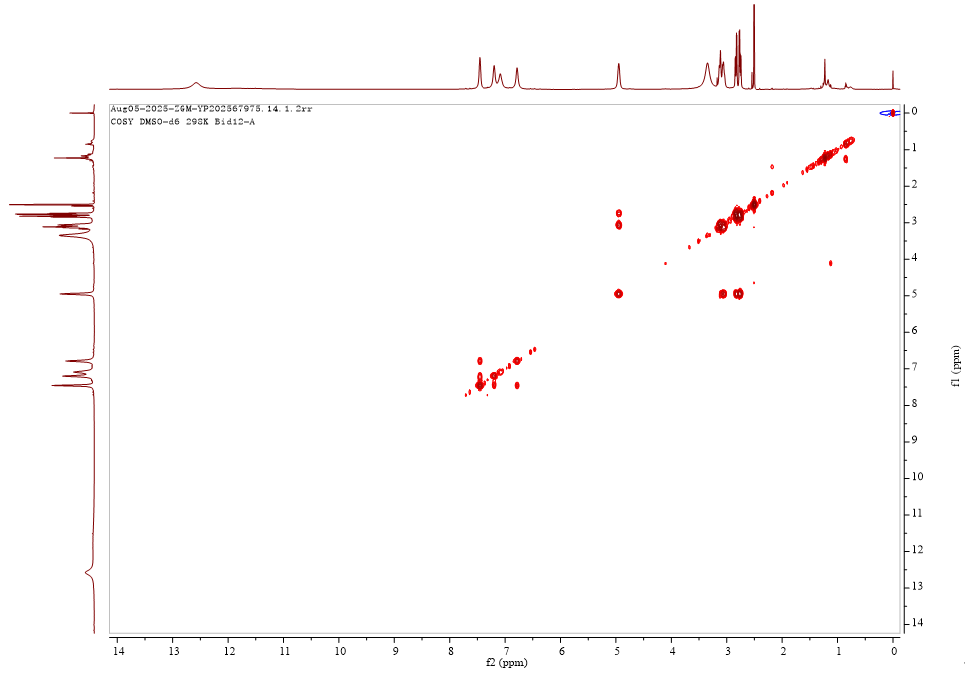


**Figure S23**. ^1^H-^1^H COSY spectra of streptoketide C in DMSO-*d*_6_


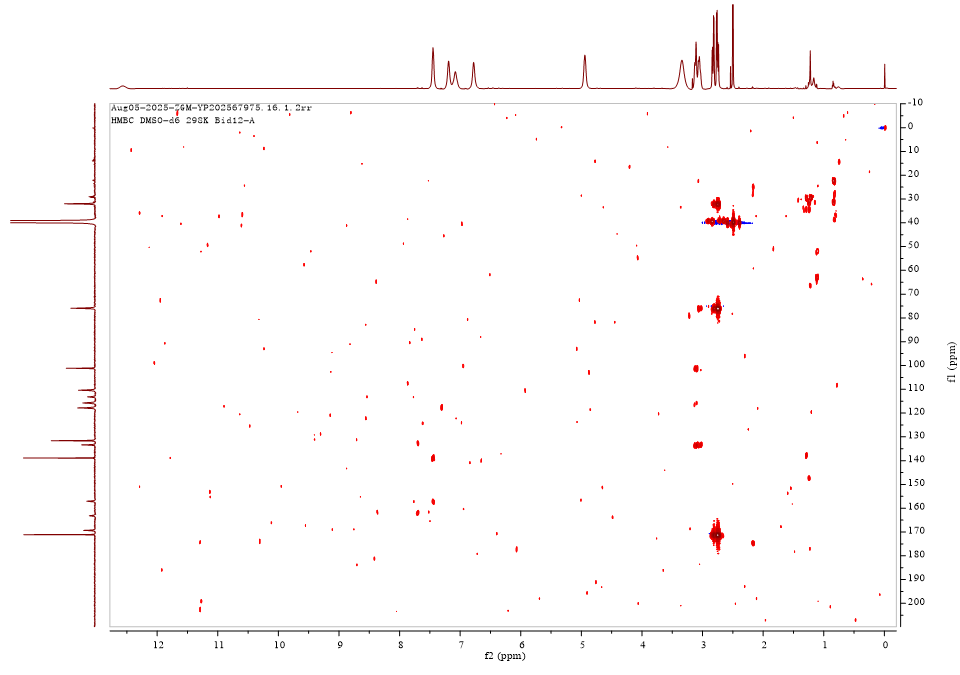


**Figure S24**. HMBC spectra of streptoketide C in DMSO-*d*_6_


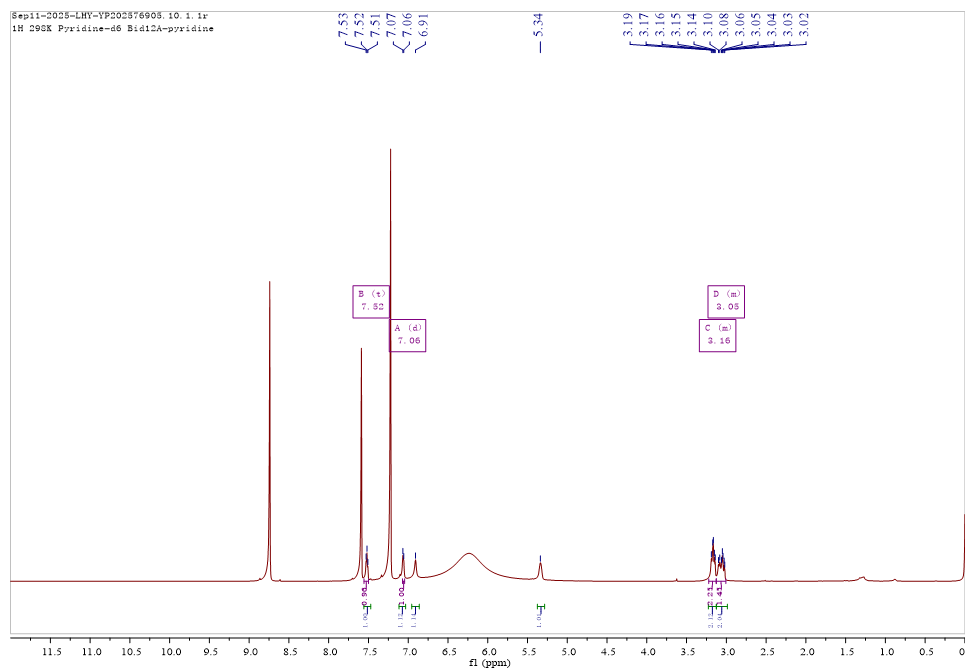


**Figure S25**. ^1^H NMR (700 MHz) spectra of streptoketide C in pyridine-*d*_5_


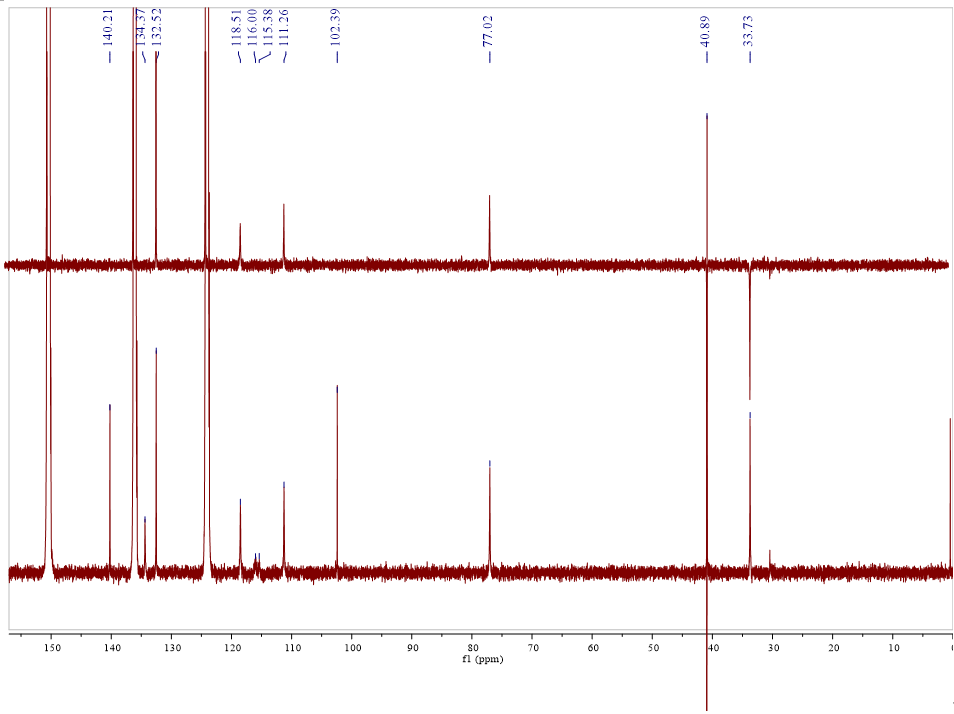


**Figure S26**. ^13^C NMR and DEPT 135° NMR (175 MHz) spectra of streptoketide C in pyridine-*d*_5_


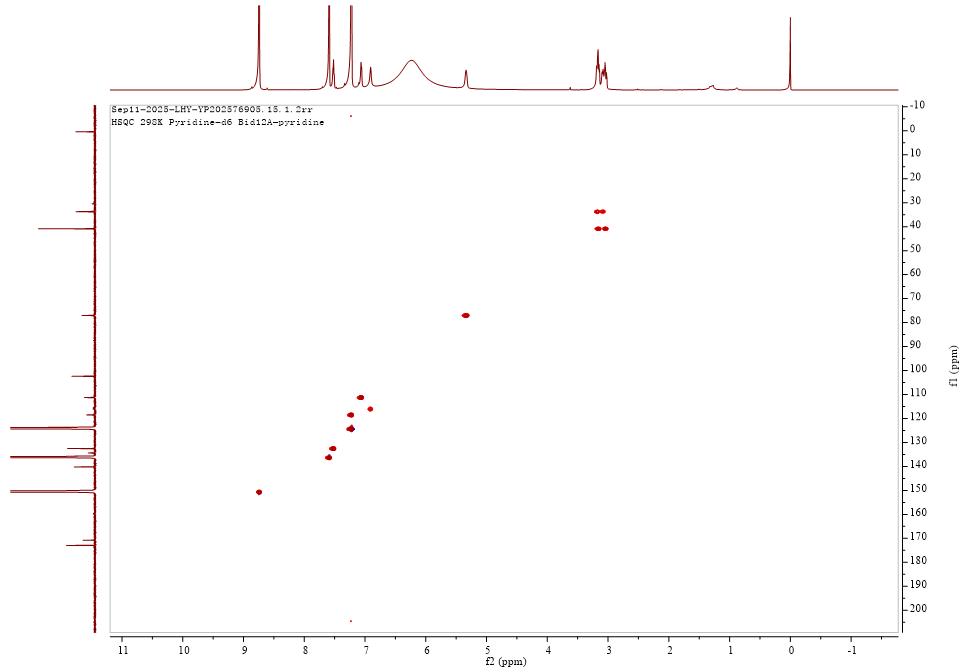


**Figure S27**. HSQC spectra of streptoketide C in pyridine-*d*_5_


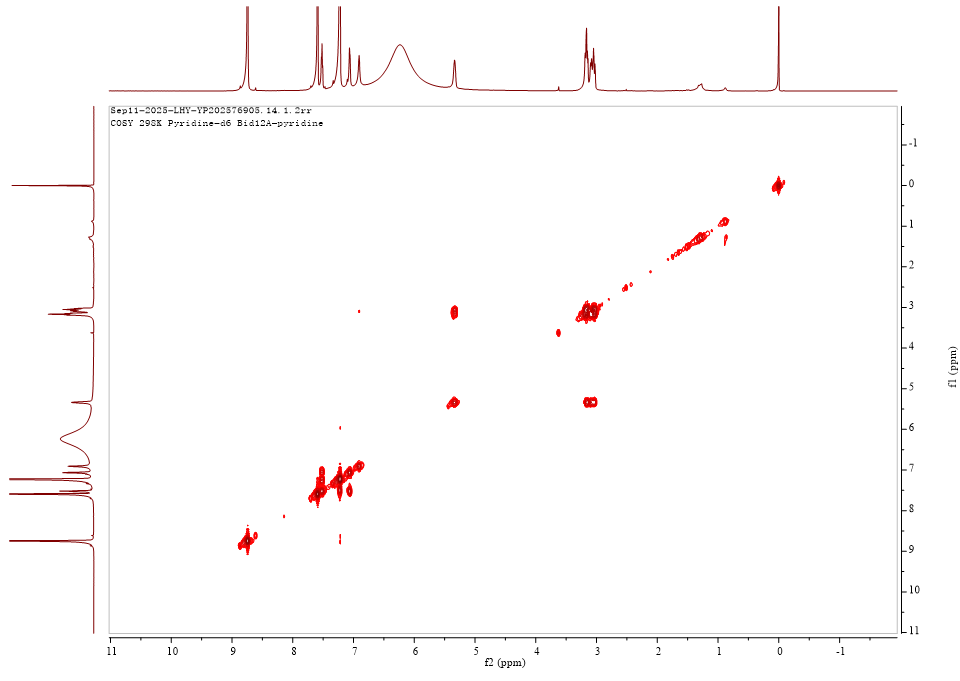


**Figure S28**. ^1^H-^1^H COSY spectra of streptoketide C in pyridine-*d*_5_


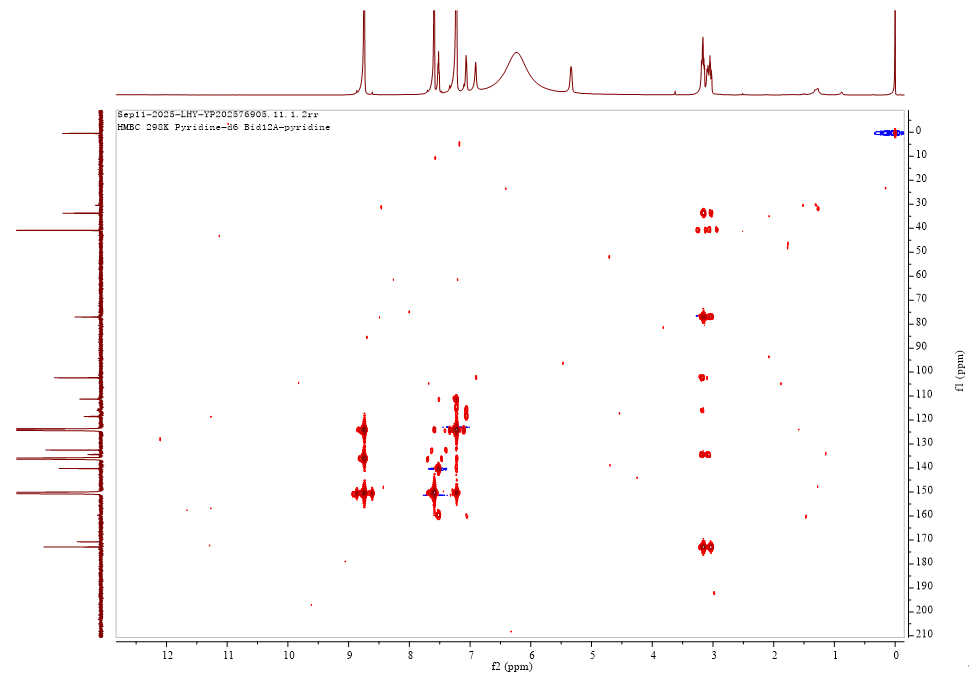


**Figure S29**. HMBC spectra of streptoketide C in pyridine-*d*_5_


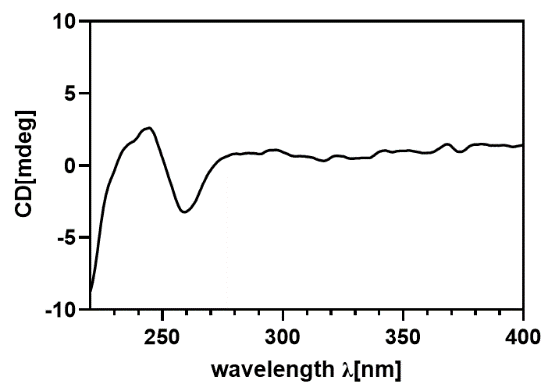


**Figure S30**. Experimental electronic circular dichroism (ECD) spectra of streptoketide C (**1b**). Comparison of the ECD spectra reported by Qian *et al* [12] for compound **1b** reveals that the C13 configuration is *S*.

**References**

[1] Guo W, Xiao Z, Huang T, Zhang K, Pan H, Tang GL, et al. Identification and characterization of a strong constitutive promoter *stnYp* for activating biosynthetic genes and producing natural products in *streptomyces*. Microb Cell Fact 2023;22(1):127-141. https://doi.org:10.1186/s12934-023-02136-9.

[2] Wang W, Li X, Wang J, Xiang S, Feng X, Yang K. An engineered strong promoter for *streptomycetes*. Appl Environ Microbiol 2013;79(14):4484-4492. https://doi.org:10.1128/AEM.00985-13.

[3] Peng Q, Gao G, Lu J, Long Q, Chen X, Zhang F, et al. Engineered *Streptomyces lividans* Strains for Optimal Identification and Expression of Cryptic Biosynthetic Gene Clusters. Front Microbiol 2018;9:3042-3057. <https://doi.org:10.3389/fmicb.2018.03042>.

[4] Gust B, Challis GL, Fowler K, Kieser T, Chater KF. PCR-targeted *Streptomyces* gene replacement identifies a protein domain needed for biosynthesis of the sesquiterpene soil odor geosmin. Proc Natl Acad Sci U S A 2003;100(4):1541-1546. <https://doi.org:10.1073/pnas.0337542100>.

[5] Xu Y, Tao F, Ma C, Xu P. New constitutive vectors: useful genetic engineering tools for biocatalysis. Appl Environ Microbiol 2013;79(8):2836-2840. https://doi.org:10.1128/AEM.03746-12.

[6] Liu G, Liu Q, Song X, Jiao X, Zhou W, Kang Q, et al. Enhancement of Acarbose Production in *Actinoplanes* sp. QQ-12 via Multiple Engineering Strategies. J Agric Food Chem 2025;73(21):12845-12855. https://doi.org:10.1021/acs.jafc.5c00613.

[7] Li J, Mu X, Dong W, Chen Y, Kang Q, Zhao G, et al. A non-carboxylative route for the efficient synthesis of central metabolite malonyl-CoA and its derived products. Nature Catalysis 2024;7(4):361-374. https://doi.org:10.1038/s41929-023-01103-2.

[8] Huang X, Xu X, Zhou L, Ma C, Wang W, Li C, et al. Naphpyrones A-H, Antibacterial Aromatic Polyketides Isolated from the Streptomyces coelicolor A3(2)/spi1 DeltaspiH3. J Agric Food Chem 2025;73(1):541-548. <https://doi.org:10.1021/acs.jafc.4c09101>.

[9] Huang S, Li N, Zhou J, He J. Development and Application of a Bacterial Artificial Chromosome (BAC) Vector for Cloning and Heterologous Expression of Large Genomic DNA Fragments in *Streptomyces*. Acta Microbiol Sin 2012;52(01):30-37. <https://doi.org:10.13343/j.cnki.wsxb.2012.01.012>.

[10] Blin K, Shaw S, Augustijn H, Reitz Z, Biermann F, Alanjary M, et al. antiSMASH 7.0: new and improved predictions for detection, regulation, chemical structures and visualisation. Nucleic Acids Res 2023;51(W1):W46-W50. https://doi.org:10.1093/nar/gkad344.

[11] Ting CP, Funk MA, Halaby SL, Zhang Z, Gonen T, van der Donk WA. Use of a scaffold peptide in the biosynthesis of amino acid-derived natural products. Science 2019;365(6450):280-284. <https://doi.org:10.1126/science.aau6232>.

[12] Qian Z, Bruhn T, D'Agostino P, Herrmann A, Haslbeck M, Antal N, et al. Discovery of the Streptoketides by Direct Cloning and Rapid Heterologous Expression of a Cryptic PKS II Gene Cluster from *Streptomyces* sp. Tu 6314. J Org Chem 2020;85(2):664-673. https://doi.org:10.1021/acs.joc.9b02741.
